# Spectral cytometry of rheumatoid arthritis patients implicates myeloid dendritic cells and granular HLA-DR+CD15+CD16+ cells in pro-inflammatory antigen presentation

**DOI:** 10.3389/fimmu.2025.1596609

**Published:** 2025-07-09

**Authors:** Christian Geier, Haani Qudsi, Estelle Khairallah, Jihad Ben Gabr, Robert Winchester, Andras Perl

**Affiliations:** ^1^ Division of Rheumatology and Clinical Immunology, Department of Medicine, State University of New York Upstate Medical University, Syracuse, NY, United States; ^2^ Norton College of Medicine, State University of New York Upstate Medical University, Syracuse, NY, United States; ^3^ Division of Rheumatology and Clinical Immunology, Department of Medicine, Columbia University, New York, NY, United States

**Keywords:** rheumatoid arthritis, CD1c+ dendritic cells, intermediate monocytes, myeloid cells, low-density granulocytes, antigen-presenting cell

## Abstract

**Introduction:**

Rheumatoid arthritis (RA) is a systemic autoimmune disease that leads to inflammation of synovial joints and other organs. Many RA patients “share” a common peptide sequence within the HLA-DR (MHC II) molecule expressed on antigen-presenting cells (APC), suggesting that HLA-DR+ cells are important in RA inflammation. We use HLA-DR positivity to comprehensively immunophenotype APC by spectral cytometry.

**Methods:**

We measured mean fluorescence intensities (MFI) of HLA-DR and molecules associated with dendritic cells (CD141, CD1c, CD163, CD11c, CD123, and CD303), monocytes (CD14 and CD16), granulocytic markers (CD15 and CCR3), co-stimulatory molecules (CD86 and CD275), and chemokine receptors (CCR2, CCR3, and CCR7) from RA patients and healthy donors by spectral flow cytometry.

**Results:**

DC2 (CD1c+) showed higher CD86, CD275 (ICOS-L), CD56, and CCR7 in RA (all *p* < 0.05). CD56 was also increased in (CD163+) DC3 (*p* = 0.0453). CD15 was increased throughout RA dendritic cell subsets and classical and intermediate monocytes (all *p* < 0.01). Except for B cells, HLA-DR was not different in RA. A distinct HLA-DR+CD15+CD16+ population appeared in RA (*p* = 0.0004), which contributed a mean of 1.3% (± SD 2.85%) to the overall HLA-DR+ APC compartment. This HLA-DR+CD15+CD16+ subset was positive for CD83, CD275, and, like plasmacytoid pDC, CD303+. However, in contrast to pDC, it formed distinct t-SNE clusters and differed from reference pDC (CD123+CD303+) by much less CD123 (*p* < 0.01). The HLA-DR+CD15+CD16+ phenotype correlated with clinical markers of systemic inflammation (*p* < 0.01).

**Discussion:**

In conclusion, dendritic cell and monocyte alterations in RA include an increased co-stimulatory phenotype of CD1c+ DC2 and CD163+ DC3 with increased CD56 and CD15 in dendritic cells and monocytes. Moreover, the blood of RA patients contains HLA-DR+ cells with shared dendritic cell and granulocytic features. These phenotypic characterizations of RA patients implicate CD1c+ DC2 and CD163+ DC3 in the systemic autoimmune disease rheumatoid arthritis and suggest that increased HLA-DR+ phenotypes with shared granulocytic and dendritic cell features can contribute to RA, potentially by providing enhanced co-stimulatory presentation of self-antigen(s) to CD4+ T lymphocytes.

## Introduction

In rheumatoid arthritis, aberrant lymphocytes can damage synovial joints and other organs (1). Antigen-presenting cells (APC) can activate lymphocytes and are considered critical to initiate immune responses (2). The profound HLA association of the HLA-DR “shared epitope” (SE) motif with RA (3, 4) led us to reason that (HLA-DR+) APC are important in RA. We hypothesized that compared with healthy controls (HC), the blood of RA patients contains HLA-DR+ APC with increased inflammatory potential (↑HLA-DR expression, a co-stimulatory phenotype; impaired inhibitory marker expression) and enhanced chemokine receptor expression. We also reasoned that through RA inflammatory conditions (such as raised levels of IFN‐γ, IL-6, and other cytokines), RA peripheral blood mononuclear cells (PBMC) may contain HLA-DR+ phenotypes (putative APC) of pathophysiologic relevance other than those meeting conventional dendritic cell and monocyte definitions. Here we use HLA-DR positivity to comprehensively immunophenotype APC by spectral cytometry, including HLA-DR+ cells (potential APC) not meeting the standard definitions for lymphocytes, monocytes, and dendritic cells (DC) from RA patients and healthy controls.

## Materials and methods

### Collection and isolation of peripheral blood mononuclear cells

We collected heparinized whole blood from RA patients meeting the 2010 ACR/EULAR classification criteria (5) and healthy control volunteers by venipuncture. The collection was approved by the institutional review board of SUNY Upstate Medical University (Syracuse, USA). Peripheral blood mononuclear cells (PBMCs) were isolated by Ficoll density gradient separation on the day of collection. PBMCs from the resulting monolayer were carefully aspirated by pipetting, washed twice in phosphate-buffered saline (PBS) with centrifugation at 350 g for 7 min at room temperature, and then resuspended in heat-inactivated fetal bovine serum (FBS) or, for some experiments, in RPMI-1640 medium supplemented with 10% FBS. We cryopreserved PBMCs by dropwise addition of a mixture of 10% dimethyl sulfoxide (DMSO) and 90% FBS to gradually achieve a final concentration of 5% DMSO. The cells were then cooled in a MrFrosty™ freezing container (ThermoFisher, Waltham, USA) at a rate of approximately -1°C per minute to -80°C and—if timely analysis was feasible—kept at -80°C or, when necessary, transferred into the vapor phase of liquid nitrogen (LN2) for long-term storage.

### Recruitment of RA patients and healthy control donors

We collected demographic and clinical information including age, gender, smoking status, rheumatoid factor (RF), and anti-cyclic citrullinated peptide (CCP) antibody status (**Supplementary Table S1**). As controls, we recruited healthy volunteers for the collection of peripheral blood and basic demographic information.

### Designation of index patients

We screened RA donors for patients with severe polyarticular synovitis and—prior to the analyses—designated the three patients with the highest severity as “index patients” (**Supplementary Table S2**). The three index patients were included in the downstream statistical analyses.

### Technical reference samples from healthy blood donors

In addition to biological controls, for some experiments, we used reference single healthy donor samples. We purchased buffy coats from healthy blood donors (New York Blood Center, New York, USA). PBMCs from buffy coats were isolated by Ficoll density gradient separation as described above but were centrifuged and washed three times in PBS at room temperature. Aliquots of single-donor PBMCs were stored in LN2 for use as technical reference controls over the course of the study.

### Spectral flow cytometry

#### Panel design

The spectral cytometry panel for antigen-presenting cells (**Supplementary Table S3**) consisted of the following: from BD Biosciences (Franklin Lakes, USA)—CD16-BUV496 (clone 3G8), CD56-BUV737 (NCAM16.2), CD45RA-BUV395 (HI100), HLA-DR-V500 (G46-6), and CD141-BB515 (1A4); from BioLegend (San Diego, USA)—CD123-BV510 (6H6), CCR7-BV421 (G043H7), CD19-PerCP-Cy5.5 (HIB19), CD14-SparkBlue550 (63D3), CD45-PerCP (2D1), CCR2-PE-Cy7 (K036C2), CD303-APC-Fire750 (201A), CD1c-AF647 (L161), CD83-PE-Cy5 (HB15e), CD86-BV711 (IT2.2), and CD155-PE/Dazzle594 (SKII.4); from ThermoFisher (Waltham, USA)—CD3-PerCP-Cy5.5 (SK7), CD11c-eFluor450 (3.9), and for viability staining—Live/Dead Fix Blue or propidium iodide (PI). For some experiments, the following additional reagents were used: CD15-BV605 (W6D3), CCR3-BUV805 (5E8), and CD19-SparkNIR 685 (HIB19). During panel development, we calculated staining indices to inform optimal concentrations and staining conditions. The selection of fluorochromes was based on a previously validated spectral cytometry panel design (6) which we modified for the purpose of this study. For all reagents, we confirmed the robust discrimination of fluorochrome/antibody by signal-to-noise ratio in single stain and multicolor “cocktail” staining pilot experiments. For most reagents, 1 μL of conjugate per sample was selected as the most suitable and was added; for the following reagents, 2 μL was added: CCR7-BV421 (G043H7), CD11c-eFluor450 (3.9), CD19-PerCP-Cy5.5 (HIB19), and CD155-PE/Dazzle594 (SKII.4).

#### Sample preparation for flow cytometry

Spectral flow cytometry of RA and healthy donor samples was performed in an interleaved fashion, in batches of four to six samples, and processed in parallel. PBMC from two samples at a time were retrieved from -80°C or LN2 storage and rapidly thawed in a 37°C water bath, followed by dropwise addition of warm complete RPMI. This was repeated either once (when four samples were analyzed) or twice for six samples. PBMCs were then washed in complete RPMI and centrifuged at 350 g for 7 min at room temperature. Cell viability following thawing was assessed by trypan blue exclusion and typically exceeded 90%.

A cocktail of fluorochrome/antibody conjugates was prepared immediately prior to staining. Following the addition of the antibody cocktail, PBMC, typically isolated from 5 mL of peripheral blood, were incubated for 30 min at room temperature in the dark in a total staining volume of approximately 50 µL. PBS supplemented with 0.5% or 1% bovine serum albumin (BSA) was used as a staining buffer (FACS buffer). Based on the results of optimization experiments, the stained cells were washed once in FACS buffer and resuspended in 125 µL of FACS buffer. For viability staining, we used Live/Dead Blue (ThermoFisher) according to the instructions provided by the manufacturer or, alternatively, added PI immediately prior to acquisition.

### Data acquisition

Flow cytometry experiments were conducted on a Cytek Aurora^®^ 5-laser spectral cytometer (Cytek Biosciences, Fremont, USA) with a laser configuration as recommended by the manufacturer.

### Bi-axial gating strategy for antigen-presenting cells

FCS Express Versions 6 and 7 (DeNovo Software, Pasadena, USA) and SpectroFlo Version 3.0.3 (Cytek Biosciences) were used for bi-axial gating of non-lymphoid antigen-presenting cells (defined as HLA-DR+[CD3/CD19]-). The forward/side scatter (FSC/SSC) gates were set generously to include the area of overlap between the larger lymphoid and smaller monocyte/DC populations (**Supplementary Figures S1A**, **S2A**). Lymphoid cells (CD3+ and CD19+), CD56+ NK cells, and other HLA-DR-negative cells were gated out (**Supplementary Figures S1A, B**, **S2A, B**).

We confirmed that enlarging the FSC/SSC gate further would not significantly improve the identification of non-lymphoid reference APC (monocytes and dendritic cells; **Supplementary Figure S3A–D**).

We gated monocytes based on combinations of CD14/CD16 as classical (CD14hiCD16lo), non-classical (CD14-CD16lo), or intermediate monocytes (CD14hiCD16+) (**Supplementary Figures S1C, S2C**) and dendritic cells as CD123+CD303+ plasmacytoid (pDC; **Supplementary Figures S1E**, **S2E**), CD141+ (DC1; **Supplementary Figures S1F, S2F** [blue gate]), and CD1c+ (DC2; **Supplementary Figures S1F, S2F** [orange gate]). We also gated the recently reported CD163+CD1c+ DC definition (DC3, **Supplementary Figures S1G, S2G** [green gate]). For better readability, we refer to all the cell populations of interest with their abbreviated designations listed in **Supplementary Table S4**.

Following the finding of increased CD15 in the reference APC populations, we quantified and characterized CD15+CD16+ granulocytic cells, including those with APC potential (HLA-DR+; **Supplementary Figures S4, S5**). Like in the original gating strategy, lymphoid cells (CD3+ and CD19+), CD56+ NK cells, and other HLA-DRneg/lo cells were gated out (**Supplementary Figures S4C, S5C**; gray gates).

CD56 can also be expressed by non-NK cells including APC (7). We thus retained those HLA-DR+ cells expressing CD56 (**Supplementary Figures S4C, S5C**; red gates). The gate boundaries were set using fluorescence minus one (FMO) controls (**Supplementary Figures S6, S7**).

All MFI read-outs were obtained from the entirety of the gated cell populations, without manually setting MFI cutoff thresholds for the reported parameters. To allow the complete assessment of technical aspects, we reported MFI measurements for all markers, regardless of biological significance in the **Supplementary Appendix**.

We reported the gated populations as % APC (HLA-DR+CD3-CD19-) and %live leukocytes. We estimated absolute cell concentrations per milliliter of blood by dividing the respective cell counts acquired during the cytometry experiments corrected for an estimated 55% yield (to account for cell loss during sample processing) by the sample volume and multiplied this value with 5 mL, the approximate volume of blood used to prepare each analyzed sample.

### T-distributed stochastic neighbor embedding

For dimensionality reduction, we applied a t-distributed stochastic neighbor embedding (t-SNE) algorithm (8). We used the Barnes–Hut implementation in FCS Express Version 7 (DeNovo Software, Pasadena, USA). We applied the following parameters: perplexity, 100; iterations, 500; seed, 6; the resolution for the resulting plots was 512 × 512 with smoothing 0–1. The color levels for the parameters represented in the t-SNE overlays were set uniformly and based on the dynamic range of a healthy control donor for each parameter used. We annotated the resulting embeddings in FCS Express for those clusters corresponding to monocytes and dendritic cells and those that did not correspond to these gating paradigms.

### Spanning-tree progression analysis of density-normalized events

To inform gating thresholds of CD56+ NK cells that distinguish them from CD56+ monocytes, we performed Spanning-tree Progression Analysis of Density-normalized Events (SPADE) analyses (9). We used the built-in SPADE function of FCS Express Version 7 (DeNovo Software, Pasadena, USA) with the following parameters: down-sampling algorithm of target density with a value of 0.2, sample size of 1,000, alpha of 5, minimum density of 0.01, local density method exact of five to six clusters (five in RA and six in HD), 0.1 minimum cells per cluster, 100 maximum iterations, seed of 6, graph layout method arch, and parameters used in transformation: CD14, CD16, CD56, and HLA-DR. We annotated the resulting tree in FCS Express for those clusters corresponding to classical monocytes, non-classical monocytes, NK cells, and those not corresponding to these gating paradigms. The node size represented the total number of events in each population.

Following t-SNE and SPADE screens, we used traditional bi-axial gating for confirmation and immunophenotyping by MFI of the identified populations.

### Statistical analysis

We used two-sided *t*-tests or Kruskal–Wallis testing to determine significant differences between RA and healthy individuals for APC subsets; a threshold of *p* < 0.05 was considered significant. We correlated the CD15+CD16+ and HLA-DR+CD15+CD16+ populations with the inflammatory markers erythrocyte sedimentation rate (ESR) and serum C-reactive protein (CRP). We preplanned comparisons of APC with plausible biological significance (HLA-DR+) between RA and healthy donors and reported all comparisons.

## Results

### The antigen-presenting capacity (HLA-DR) is not uniformly increased in RA APC

**Figure 1:**
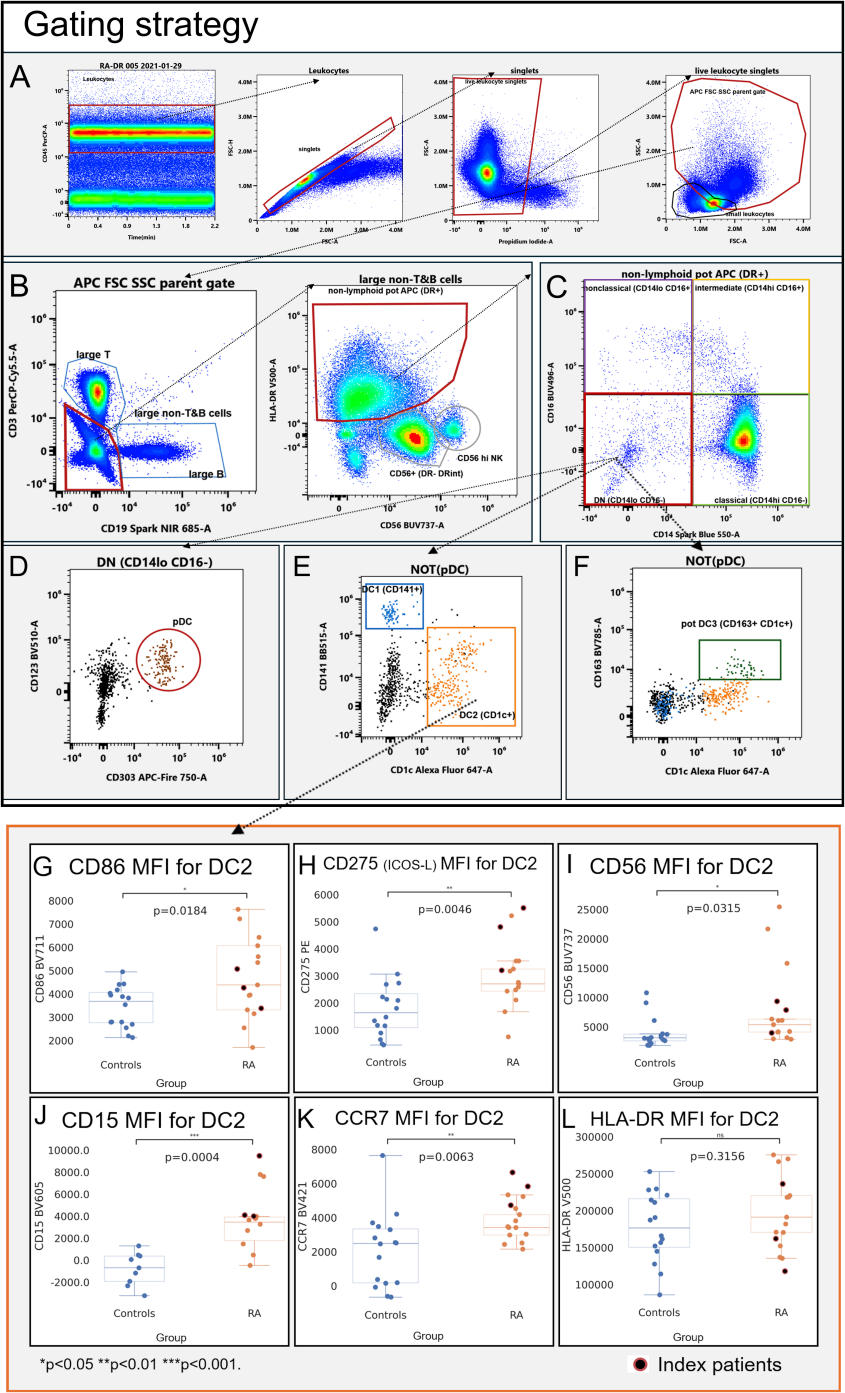
Gating strategy **(A–F)** and immunophenotype **(G–L)** of CD1c+ dendritic cells (DC2; CD1c+). Mean fluorescence intensities (MFI) were measured by spectral cytometry (y-axis). RA DC2 are characterized by higher MFI for CD56 **(A)**, CD15 **(B)**, the co-stimulatory molecules CD86 and CD275 (ICOS-L) **(C, D)**, and C–C chemokine receptor type 7 **(E)**. HLA-DR MFI did not differ between RA and controls **(F)**. Blue, healthy control donors; orange, RA patients. RA index patients are highlighted in red.

Given the importance of HLA-DR as a molecular basis for antigen presentation, we compared the HLA-DR mean fluorescence intensities (MFI) of RA APC with healthy control donors (HC) using the gating strategy in **Figures 1A–F**. In RA B cells (CD19+), HLA-DR was increased, as expected (**Supplementary Figure S8A**). Unexpectedly, HLA-DR for dendritic cells (DC1, DC2, plasmacytoid DC, and the recently described DC3 (10, 11)) and monocytes (classical, intermediate, and non-classical) did not differ between RA and HC (**Supplementary Figures S8B–G**; *p* > 0.05), nor was there any significant increase in the frequencies of these APC subsets (**Supplementary Figures S9A–G, S10A–G**; *p* > 0.05).

We thus focused on the non-HLA-DR features of non-lymphoid (CD3-CD19-) APC, immunophenotyping dendritic cells and monocyte subsets.

### Myeloid dendritic cells exhibit a co-stimulatory phenotype in RA

DC2 (CD1c+; **Supplementary Figure S11**)—the most abundant dendritic cell subset and considered to be the most potent APC (12)—had higher MFIs of the co-stimulatory molecules CD86 (**Figure 1G**, p = 0.0184) and CD275 (ICOS-L; **Figure 1H**, *p* = 0.0046), consistent with an increased capacity of RA DC2 to provide co-stimulation to T cells. RA DC2 also had increases of CD56 (**Figure 1I**, p = 0.0315) and CD15 (**Figure 1J**, *p* = 0.0002), consistent with an increased adhesive propensity (13). C–C chemokine receptor type 7 (CCR7) was higher in RA DC2, consistent with an increased migratory potential to lymphatic tissues (**Figure 1K**, *p* = 0.0063).

DC1 (CD141+: **Supplementary Figure S12**) showed significant increases in CD275 (ICOS-L) in RA (**Supplementary Figure S12U**; *p* = 0.0065). CCR7 was also higher compared to control DC1 (**Supplementary Figure S12D**; *p* = 0.0001), but in contrast with DC2, CD86 did not differ (**Supplementary Figure S12P**; *p* = 0.6395). We also observed higher CD15 in RA DC1 (CD141+) (**Supplementary Figure S12I**, *p* = 0.0002); CD56 was numerically higher but not statistically different (**Supplementary Figure S12N**, *p* = 0.1321).

We also gated the recently proposed DC3 definition (CD1c+CD163+; **Supplementary Figure S13**) (10, 11). Like DC1 and DC2, DC3 showed clearly increased CD15 (**Supplementary Figure S13I**, *p* = 0.0001) and CD56 (**Supplementary Figure S13N**, *p* = 0.0453) as well as CD275 (**Supplementary Figure S13U**, *p* = 0.0018), whereas CD86 did not differ (**Supplementary Figure S13P**, *p* = 0.2876).

In plasmacytoid dendritic cells (CD123+CD303+ pDC; **Supplementary Figure S14**), CD15 increases were comparatively subtle (**Supplementary Figure S14I**, *p* = 0.0432); CD56 did not differ (*p* = 0.0774) and pDC changes in RA were otherwise largely confined to increased CCR7 (**Supplementary Figure S14D**, *p* = 0.0337) and a low-level expression of CD275 (**Supplementary Figure S14U**, *p* = 0.0220).

### Increased CD56 in dendritic cells is not due to NK cells

**Figure 2:**
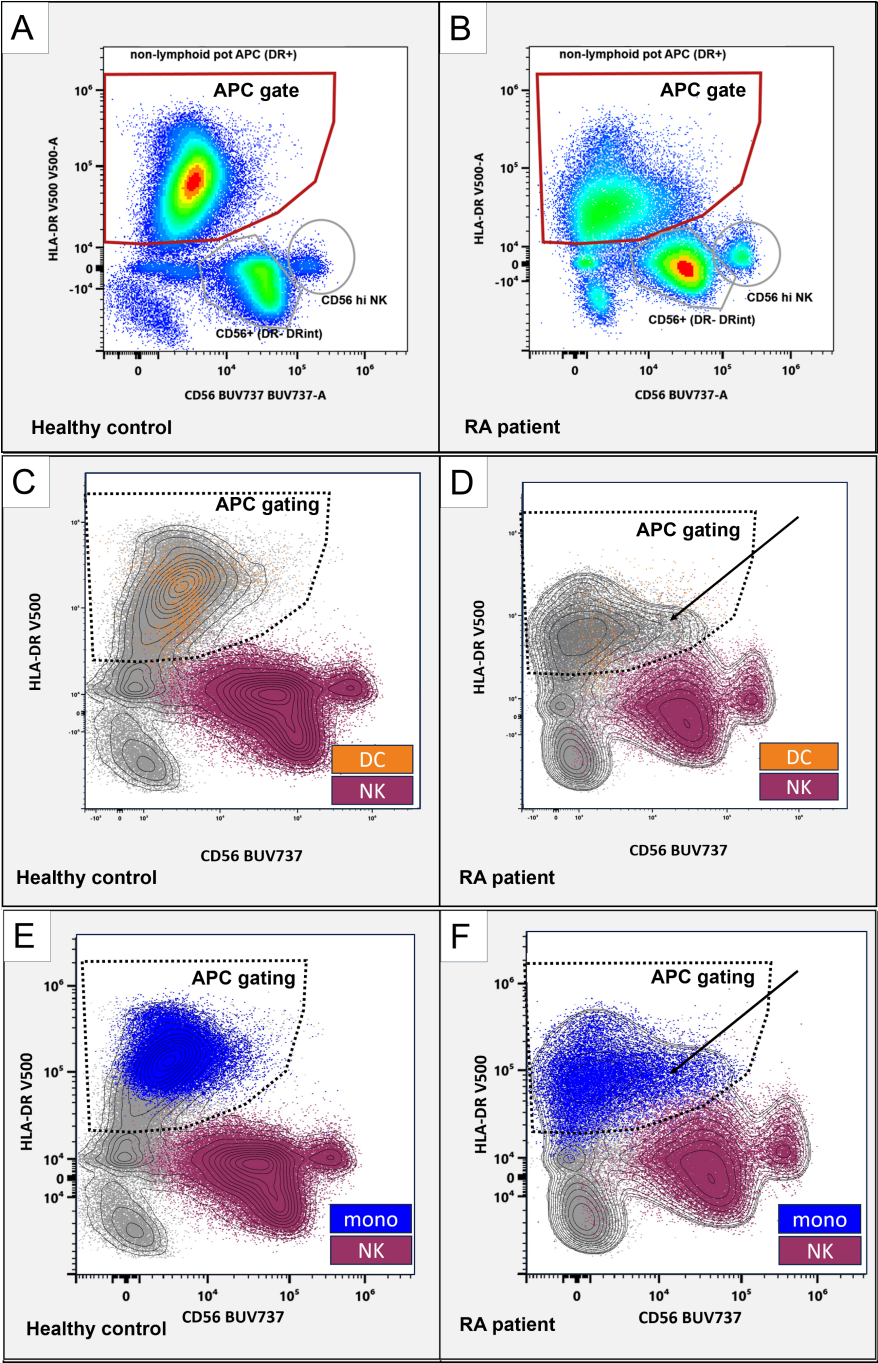
CD56 in APC and NK cell populations. Representative flow cytometry plots, gated on non-lymphoid live singlets (CD3-CD19-). CD56 (x-axis) and HLA-DR (y-axis). Gated are HLA-DR+ antigen-presenting cells (red gates; **A**, **B**). Distinct CD56+ NK cells are marked with gray circles. **(C-F)**: NK cells (purple) and monocytes (blue) classified using the SPADE algorithm and dendritic cells (orange) were backgated as color-coded dot plots and manually overlayed with a contour view representation showing increased CD56 APC (solid arrows) and the APC gating strategy (dashed borders). CD56 (x-axis) and HLA-DR (y-axis). Left panels, healthy control donor. Right panels, RA patient.

**Figure 3:**
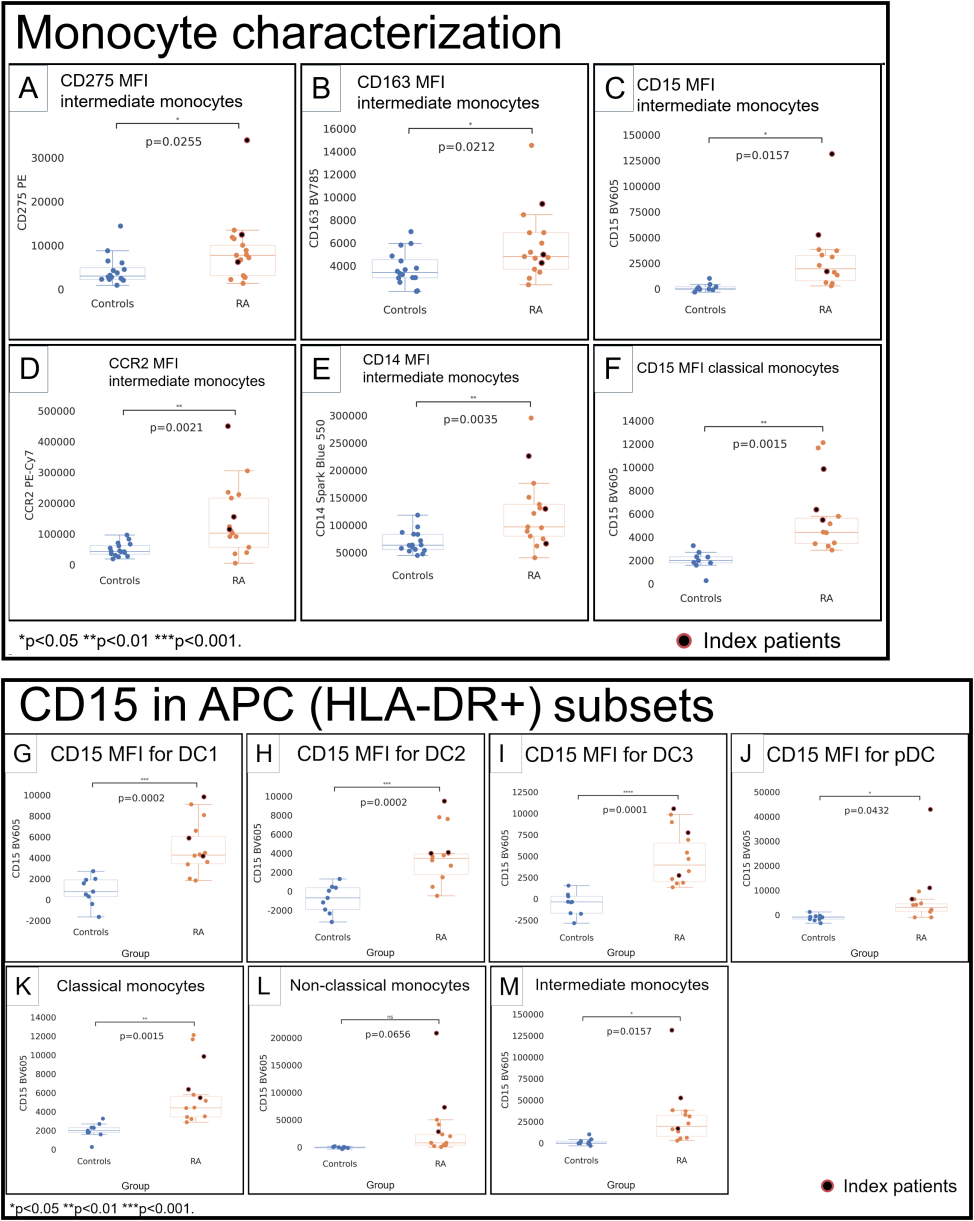
Monocytes **(A–F)** and CD15 in APC subsets **(G–M)** in healthy control donors (blue) and RA patients (orange). Mean fluorescence intensities (MFI) were measured by spectral cytometry (y-axis). RA intermediate monocytes differed by higher MFI for CD275 **(A)**, CD163 **(B)**, CD15 **(C)**, C–C chemokine receptor type 2 **(D)**, and CD14 **(E)**. Classical monocytes had higher CD15 **(F)**. CD15 in DC1 **(G)**, DC2 **(H)**, DC3 **(I)**, pDC **(J)**, classical monocytes **(K)**, non-classical monocytes (**L**), and intermediate monocytes **(M)**. RA index patients are highlighted separately (red).

Given the association of CD56 with natural killer (NK) cells, we examined whether the shift to higher CD56 in DC2 and DC3 in RA could be due to the inclusion of NK cells, as NK cells have been reported to express HLA-DR under inflammatory conditions (14, 15). During the gating of healthy donors, NK cells formed two distinct CD56-positive populations with much lower levels of HLA-DR, allowing their separation from the APC gate (**Figure 2A**, gray gates and red gate). RA patients showed higher CD56 in APCs (**Figure 2B**, red gate). While resulting in closer proximity of APC and NK cell gates, the events in the red APC gate–used for all downstream analyses–remained clearly distinguishable (**Figure 2B** as well as **Supplementary Figures S1, S2**) with the contour overlay supporting that the CD56+HLA-DR+ population in RA originates from APC rather than NK cells (**Figures 3D, F**, black arrows).

To further classify APC and NK cells, we used unsupervised Spanning-tree Progression Analysis of Density-normalized Events (SPADE) clustering (9)—applied on the total non-T&B cell (CD3 and CD19 negative) population—to group cells into NK cells or APC (DC and monocytes; **Supplementary Figure S15**).

Back-gating of NK and monocyte groupings created by SPADE and DC subsets (DC1, DC2, DC3, and pDC) onto the CD56/HLA-DR biaxial view showed that DC (**Figures 2C, D**, orange) and monocytes (**Figures 2E, F**, blue) are located within the APC gating strategy (**Figures 2C–F**, dashed gates), whereas NK cells (**Figures 2C–F**, purple) are outside. Collectively, this suggests that the observed CD56 increases are not attributable to the inclusion of (HLA-DR+) NK cells but, rather, represent bona fide CD56 increases in these dendritic cells. We next focused on monocyte subsets.

### Intermediate and classical monocytes show increased CD15 in RA

Intermediate monocytes (**Supplementary Figure S16**) showed higher CD275 (*p* = 0.0255; **Figure 3A**) and CD163 (*p* = 0.0212; **Figure 3B**) and had a much higher CD15 in RA (RA: 31.4K vs. HC: 1,497; *p* = 0.0157; **Figure 3C**). C–C chemokine receptor type 2 (CCR2) in intermediate monocytes was higher (*p* = 0.0021; **Figure 3D**), consistent with an increased propensity to migrate to inflammatory sites (16). CD14 was higher (*p* = 0.0035; **Figure 3E**). Classical (**Supplementary Figure S16**) and non-classical (**Supplementary Figure S17**) monocytes did not differ for most relevant surface molecules, except for higher CD15 in classical monocytes (RA: 6,022, HC: 2,003; *p* = 0.0015; **Figure 3F**).

A comprehensive numerical comparison of the immunophenotype of dendritic cell and monocyte subsets in RA is listed in **Table 1**.

**Table 1:** Immunophenotype in mean MFI (SD) of dendritic cell and monocyte subsets.

HC (SD) RA (SD) *p*-value	DC1 (CD141+)	DC2 (CD1c+)	DC3 (CD163+CD1c+)	pDC (CD123+CD303+)	Classical monocytes (CD14hi CD16lo)	Intermediate monocytes (CD14hi CD16int)	Non-classical monocytes (CD16hi CD14 lo)
% APC (HLA-DR+CD3-CD19-)	0.11 (0.11) 0.17 (0.17) *p* = 0.3750	2.17 (1.66) 2.83 (1.60) *p* = 0.1871	0.38 (0.27) 0.34 (0.27) *p* = 0.7629	2.35 (1.99) 1.62 (1.10) *p* = 0.2913	79.9 (11.0) 81.9 (12.1) *p* = 0.6242	2.86 (2.05) 4.25 (3.06) *p* = 0.2000	5.82 (4.51) 6.22 (7.23) *p* = 0.5717
% live leukocytes	0.010 (0.012) 0.018 (0.018) *p* = 0.1374	0.29 (0.24) 0.22 (0.11) *p* = 0.7201	0.04 (0.02) 0.05 (0.05) *p* = 0.9238	0.24 (0.24) 0.17 (0.12) *p* = 0.5838	9.26 (5.60) 11.87 (8.09) *p* = 0.4739	0.27 (0.16) 0.49 (0.45) *p* = 0.1518	0.50 (0.42) 0.59 (0.65) *p* = 0.9850
HLA-DR V500	125K (35.7K) 146K (42.2K) *p* = 0.1660	195K (50K) 178K (46K) *p* = 0.3156	207K (42.4K) 229K (32.9K) *p* = 0.1057	82.9K (25.4K) 100K (34.7K) *p* = 0.1158	76.9K (18.9K) 75.8K (14.5K) *p* = 0.8629	82.9K (22.3K) 92.1K (21.9K) *p* = 0.2464	48.3K (13.5K) 59.0K (16.5K) *p* = 0.0532
CD56 BUV737	6,749 (5,284) 12.4K (13.6K) *p* = 0.1321	3,966 (2,555) 8,099 (6,867) *p* = 0.0315	3,850 (1,972) 9,077 (9,812) *p* = 0.0453	4,162 (1,708) 5,810 (3,174) *p* = 0.0774	2,974 (1,590) 4,991 (6,467) *p* = 0.2350	3,762 (1,314) 4,561 (3,254) *p* = 0.3696	3,417 (759) 5,221 (3,985) *p* = 0.0855
CD15 BV605	862 (1,351) 5,195 (2,550) *p* = 0.0002	-791 (1,490) 3,987 (2,868) *p* = 0.0002	-557 (1,334) 5,193 (3,286) *p* = 0.0001	-1,149 (1,297) 7,112 (11.3K) *p* = 0.0432	2,003 (832) 6,022 (3,173) *p* = 0.0015	1,497 (3,889) 31.4K (33.5K) *p* = 0.0157	-539 (1,624) 36.2K (56.2K) *p* = 0.0656
CD14 SB550	4,739 (9,439) -1,530 (9,247) *p* = 0.0721	4,900 (2,125) 6,443 (1,833) *p* = 0.0356	9,545 (3,679) 11.8K (3,957) *p* = 0.1021	3,083 (1,876) 4,159 (2,633) *p* = 0.1931	186K (37.5K) 219K (45.6K) *p* = 0.0310	69.0K (19.9K) 123K (65.7K) *p* = 0.0035	7,776 (3,008) 8,915 (2,736) *p* = 0.2714
CD11c eFluor450	4,068 (4,027) 3,585 (3,927) *p* = 0.7379	14.9K (7,634) 22.1K (17.8K) *p* = 0.1462	20.3K (9,110) 32.8K (24.3K) *p* = 0.0644	-1,428 (1,046) -1,292 (2,155) *p* = 0.8215	12.4K (5,943) 17.4K (11.8K) *p* = 0.1414	37.5K (16.4K) 40.5K (23.5K) *p* = 0.6831	29.8K (15.7K) 39.7K (27.3K) *p* = 0.2197
CD163 BV785	1,369 (764) 1,719 (886) *p* = 0.2483	4,734 (1,611) 5,422 (1,524) *p* = 0.2252	10.4K (1,553) 10.9K (1,352) *p* = 0.3471	4,316 (1,378) 5,259 (1,689) *p* = 0.0940	5,147 (1,839) 6,081 (1,470) *p* = 0.1231	3,784 (1,477) 5,824 (3,012) *p* = 0.0212	3,370 (1,063) 4,053 (2,061) *p* = 0.2476
CD1c AF647	2,560 (1,481) 2,469 (1,481) *p* = 0.6230	54.2K (10.8K) 57.1K (21.2K) *p* = 0.6230	59.6K (16.7K) 66.4K (23.5K) *p* = 0.3503	5,662 (2,857) 5,155 (2,869) *p* = 0.6204	4,592 (2,736) 4,711 (2,525) *p* = 0.8986	4,920 (2,706) 5,371 (2,738) *p* = 0.6423	3,820 (1,998) 3,737 (1,877) *p* = 0.9035
CD45RA BUV395	42.7K (38.9K) 42.2K (30.4K) *p* = 0.9686	67.0K (37.4K) 89.8K (53.8K) *p* = 0.1743	41.2K (13.7K) 54.2K (58.0K) *p* = 0.3881	163K (28.4K) 164K (27.0K) *p* = 0.8816	26.4K (10.2K) 27.8K (10.3K) *p* = 0.6987	151K (66.4K) 90.9K (35.0K) *p* = 0.0032	250K (72.6K) 221K (77.3K) *p* = 0.2954
CD275 PE	2,482 (1,647) 4,428 (2,037) *p* = 0.0065	1,822 (1,127) 3,114 (1,253) *p* = 0.0046	2,214 (1,340) 3,750 (1,193) *p* = 0.0018	1,495 (838) 2,648 (1,715) *p* = 0.0220	6,459 (2,682) 7,711 (3,581) *p* = 0.2720	4,322 (3,353) 9,208 (7,607) *p* = 0.0255	4,408 (5,857) 10.6K (13.4K) *p* = 0.0989
CD16 BUV496	-97,73 (7,864) -3,888 (5,489) *p* = 0.0230	-5,999 (5,548) -403 (3,262) *p* = 0.0110	-7,584 (8,287) 1,432 (3,991) *p* = 0.0005	-4,558 (4,496) 1,826 (3,140) *p* = 0.0001	-4,140 (11,586) 4,697 (5,813) *p* = 0.0106	102K (46.2K) 98.0K (22.2K) *p* = 0.7541	158K (56.5K) 179K (39.4K) *p* = 0.2303
CCR2 PE-Cy7	49.1K (17.3K) 50.5K (15.3K) *p* = 0.8094	177K (51.4K) 170K (48.1) *p* = 0.6651	311K (54.3K) 297K (73.6K) *p* = 0.5499	166K (47.9K) 155K (48.3K) *p* = 0.5180	312K (60.6K) 316K (48.6K) *p* = 0.8480	48.8K (21.7K) 147K (115K) *p* = 0.0021	8,070 (7,713) 14.7K (11.5K) *p* = 0.0648
CCR3 BUV805	2,752 (864) 3,383 (1,363) *p* = 0.2354	2,751 (863) 3,383 (1,363) *p* = 0.2354	4,568 (908) 5,145 (2,673) *p* = 0.5422	2,527 (940) 2,485 (987) *p* = 0.9217	4,692 (1,288) 4,993 (1,006) *p* = 0.5461	4,439 (1,012) 5,487 (1,628) *p* = 0.1029	5,252 (928) 5,672 (1,377) *p* = 0.4360
CCR7 BV421	1,222 (1,398) 3,504 (1,474) *p* = 0.0001	2,108 (2,196) 3,986 (1,306) *p* = 0.0062	3,248 (8,690) 3,726 (1,787) *p* = 0.8308	3,119 (979) 3,957 (1,144) *p* = 0.0337	983 (1,421) 2,821 (1,520) *p* = 0.0013	3,234 (1,221) 4,746 (2,590) *p* = 0.0431	4,088 (728) 5,197 (2,167) *p* = 0.0619

Two-sided *t*-tests.

HC, healthy control donors (first row per cell); RA, rheumatoid arthritis (second row per cell); K, thousand.

These overlapping patterns of changes in dendritic cells and monocytes prompted us to re-analyze these populations jointly. We performed further analyses to screen for potential shifts in CD56 and CD15 in the HLA-DR+ APC compartment in RA.

### t-SNE screens of RA antigen-presenting cells suggest CD56 and CD15 APC signatures and a distinct HLA-DR+CD15+CD16+ population

We used t-distributed stochastic neighbor embedding (t-SNE) on pre-gated CD3-CD19- cells to visualize APC and NK cells (CD3-CD19-CD56+) from newly diagnosed RA patients experiencing active polyarthritis. In healthy control donors, APC arranged into clusters consistent with monocyte and dendritic cell subsets (**Supplementary Figure S19A**, finely dashed lines) as expected. In contrast, a t-SNE screen of RA index patient 005 (**Supplementary Table S2**) showed widely increased CD56 in RA monocytes and dendritic cells (**Supplementary Figure S20B**, solid and dashed red arrows) in addition to NK cells (**Supplementary Figure S20**, top left CD56+ population).

Regarding CD15, we saw increased CD15 in monocytes and dendritic cells (**Supplementary Figure S20F**, solid and dashed red arrows); additionally, we observed increased CD15+CD16+ cells with high side scatter (**Supplementary Figure S20A, D, F**, top right population), consistent with an expansion of low-density neutrophils (17) in this index patient. In contrast, index patient 009 (**Supplementary Table S2**) showed a different pattern of APC changes. t-SNE focusing on APC revealed a large cluster of HLA-DR+CD15+CD16+ cells as a striking feature (**Supplementary Figure S21A**, red arrow and B-J, dashed circle). This highlighted population was also positive for CD275, CD83 (**Supplementary Figures S21A-D**), and the plasmacytoid DC marker CD303 (**Supplementary Figure S21E**).

These screens suggest that some RA patients have CD15 and CD56 increases in multiple APC lineages and may have expanded HLA-DR+CD15+CD16+ phenotypes contributing to their overall APC compartment. We then examined whether such changes are present in our overall RA patient cohort.

### The RA APC compartment shows marked increases of the granulocyte-associated molecule CD15 in dendritic cells and monocytes

We quantified CD15 by MFI in dendritic cell and monocyte subsets across the patient cohort. Except for non-classical monocytes (*p* = 0.0656; **Figure 3L**), we saw increased CD15 MFI throughout dendritic cells and monocytes (*p* < 0.05; **Figures 3A–E, G**).

This suggests that CD15 increases in RA monocytes and dendritic cells can affect multiple lineages of monocyte and dendritic cells and raises the possibility of a fundamental change of CD15 in myeloid cells in the autoimmune disease RA. To clarify the relationship of CD15+ cells in RA, we then specifically focused on CD15+ phenotypes.

### Distinct HLA-DR+CD15+CD16+ granulocytic phenotypes are increased in RA and express surface molecules commonly associated with dendritic cells

**Figure 4:**
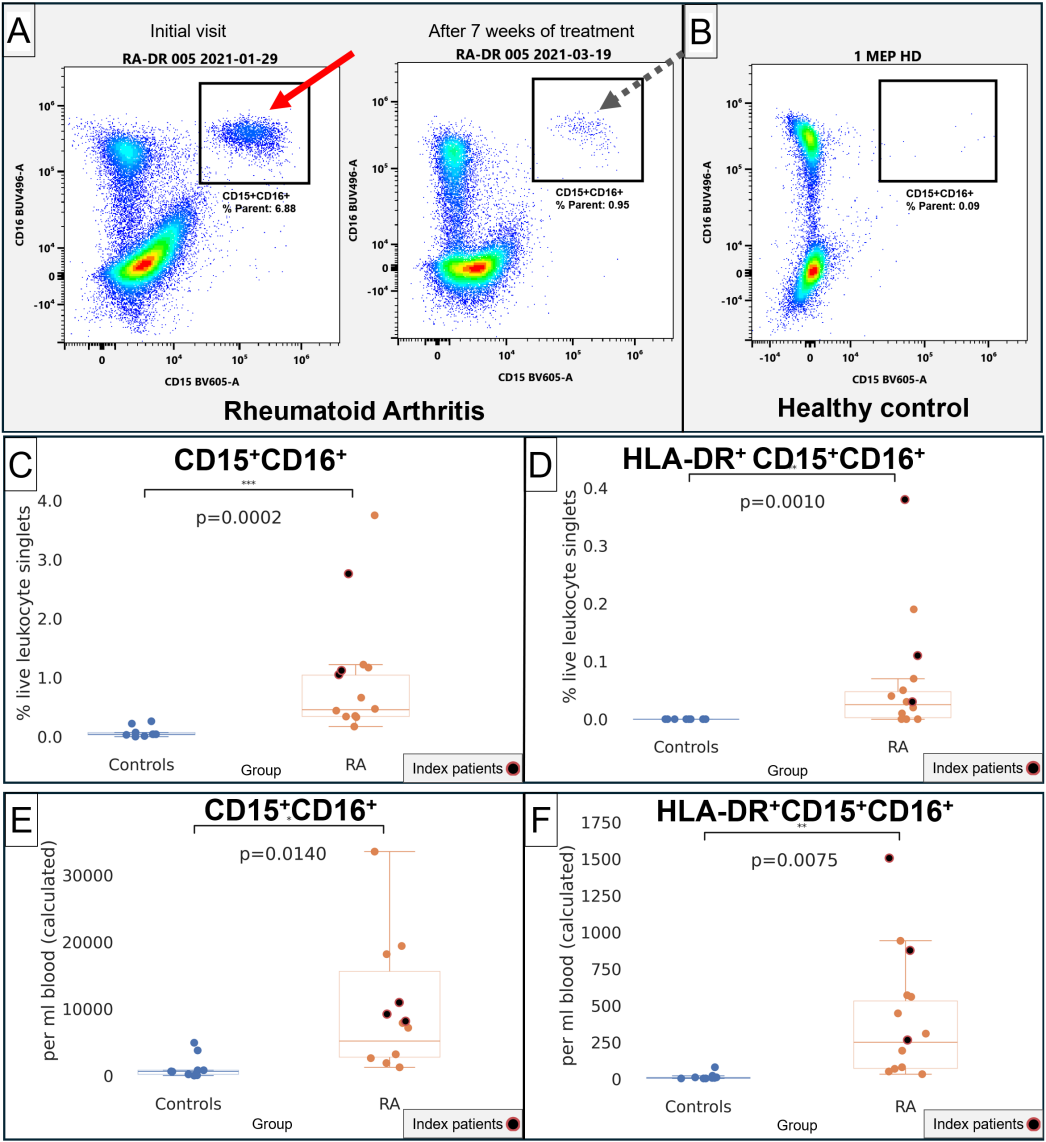
Quantification of CD15+CD16+ and CD15+CD16+HLA-DR+ in RA and healthy control donors. **(A)** Representative flow cytometry of CD15 (x-axis) and CD16 (y-axis). Left plot, index patient 005 at the initial visit at a time of severe disease. Right plot, the same patient after starting treatment. **(B)** Representative healthy control donor. **(C)** Quantification of CD15+CD16+ in healthy controls (blue) and RA patients (orange), in % live lymphocyte singlets. **(D)** Quantification of HLA-DR+CD15+CD16+ in healthy controls (blue) and RA patients (orange), in % live lymphocyte singlets. **(E)** Quantification of CD15+CD16+ in healthy controls (blue) and RA patients (orange), in cells/mL of blood. **(F)** Quantification of HLA-DR+CD15+CD16+ in healthy controls (blue) and RA patients (orange), in cells/mL of blood. For **(C–F)**, index patients are highlighted in red.

We then specifically gated CD15+CD16+ and HLA-DR+CD15+CD16+ using the gating strategy in **Supplementary Figures S4, S5**. CD15+CD16+ were much higher in RA patients (**Figure 4A**, arrows) compared with healthy controls (**Figure 4B**). CD15+CD16+ and HLA-DR+CD15+CD16+ populations were significantly increased, both when measured as a percentage of live leukocytes as well as in absolute numbers per milliliter of blood (*p* = 0.002 and *p* = 0.0010 in % live leukocytes, **Figures 4C, D**; *p* = 0.0140 and *p* = 0.0075 in cells per milliliter of blood, **Figures 4E, F**). The overall CD15+CD16+ population displayed a side scatter profile and immunophenotype consistent with low-density granulocytes; given our focus on potential antigen-presenting cells, we then specifically focused on the HLA-DR-positive subset and compared its phenotype with monocytes and dendritic cells.

The HLA-DR+CD15+CD16+ phenotype contributed a mean of 1.3% (SD 2.85%) of the total APC compartment in RA, being more abundant than DC1 (0.17%), DC3 (0.34%), and similar to pDC (1.62%) but less frequent than DC2 or any of the monocyte subsets (**Table 2**). The intensity of HLA-DR was 44.1K (SD 9954), below the dendritic cell and monocyte subsets. Consistent with the t-SNE screens, the plasmacytoid dendritic cell marker CD303 was highly positive at 72.8K (SD 42.9K), but CD123 was much lower than in pDC (**Table 2**). Notably, the HLA-DR+CD15+CD16+ phenotype was also characterized by high CD83 and CD275 (ICOS-L), which are commonly associated with dendritic cells (18, 19).

**Table 2:** Immunophenotype of HLA-DR+CD15+CD16+ in mean MFI (SD) in relationship to dendritic cell and monocyte subsets in RA patients (*n* = 16).

RA APC (SD)	HLA-DR+ CD15+ CD16+	DC1 (CD141+)	DC2 (CD1c+)	DC3 (CD163+CD1c+)	pDC (CD123+CD303+)	Classical monocytes (CD14hi CD16lo)	Intermediate monocytes (CD14hi CD16int)	Non-classical monocytes (CD16hi CD14 lo)
% APC (HLA-DR+CD3-CD19-)	1.30 (2.85)	0.17 (0.17)	2.83 (1.60)	0.34 (0.27)	1.62 (1.10)	81.9 (12.1)	4.25 (3.06)	6.22 (7.23)
% live leukocytes	0.07 (0.11)	0.018 (0.018)	0.22 (0.11)	0.05 (0.05)	0.17 (0.12)	11.87 (8.09)	0.49 (0.45)	0.59 (0.65)
Side scatter	1.53M (265K)	1.05M (210K)	907K (191K)	1.02M (159K)	845K (138K)	1.15M (223K)	1.32M (306K)	1.01M (293K)
HLA-DR V500	44.1K (9,954)	146K (42.2K)	178K (46K)	229K (32.9K)	100K (34.7K)	75.8K (14.5K)	92.1K (21.9K)	59.0K (16.5K)
CD56 BUV737	3,532 (1,770)	12.4K (13.6K)	8,099 (6,867)	9,077 (9,812)	5,810 (3,174)	4,991 (6,467)	4,561 (3,254)	5,221 (3,985)
CD15 BV605	233K (94.5K)	5,195 (2,550)	3,987 (2,868)	5,193 (3,286)	7,112 (11.3K)	6,022 (3,173)	31.4K (33.5K)	36.2K (56.2K)
CD14 SB550	73.1K (73.1K)	-1,530 (9,247)	6,443 (1,833)	11.8K (3,957)	4,159 (2,633)	219K (45.6K)	123K (65.7K)	8,915 (2,736)
CD11c eFluor450	14.9K (12.3K)	3,585 (3,927)	22.1K (17.8K)	32.8K (24.3K)	-1,292 (2,155)	17.4K (11.8K)	40.5K (23.5K)	39.7K (27.3K)
CD163 BV785	7,021 (4,591)	1,719 (886)	5,422 (1,524)	10.9K (1,352)	5,259 (1,689)	6,081 (1,470)	5,824 (3,012)	4,053 (2,061)
CD1c AF647	3,641 (2,807)	2,469 (1,481)	57.1K (21.2K)	66.4K (23.5K)	5,155 (2,869)	4,711 (2,525)	5,371 (2,738)	3,737 (1,877)
CD45RA BUV395	29.0K (29.1K)	42.2K (30.4K)	89.8K (53.8K)	54.2K (58.0K)	164K (27.0K)	27.8K (10.3K)	90.9K (35.0K)	221K (77.3K)
CD16 BUV496	200K (47.4K)	-3,888 (5,489)	-403 (3,262)	1,432 (3,991)	1,826 (3,140)	4,697 (5,813)	98.0K (22.2K)	179K (39.4K)
CCR3 BUV805	6,722 (5,579)	3,383 (1,363)	3,383 (1,363)	5,145 (2,673)	2,485 (987)	4,993 (1,006)	5,487 (1,628)	5,672 (1,377)
CCR7 BV421	5,225 (3,001)	3,504 (1,474)	3,986 (1,306)	3,726 (1,787)	3,957 (1,144)	2,821 (1,520)	4,746 (2,590)	5,197 (2,167)
CD123 BV510	14.4K (5,719)	18.9K (8,369)	21.7K (9,664)	27.1K (12.6K)	59.8K (19.6K)	1,807 (12.8K)	16.2K (12.4K)	16.3K (9,395)
CD86 BV711	7,603 (8,179)	3,232 (1,548)	4,627 (1,676)	5,245 (1,805)	2,893 (1,206)	11,942 (3,913)	18.7K (4,581)	14.3K (4,482)
CD163 BV785	7,021 (4,591)	1,719 (886)	5,422 (1,524)	10.9K (1,352)	5,259 (1,689)	6,081 (1,470)	5,824 (30,12)	4,053 (2,061)
CD141 BB515	13.4K (6,726)	514K (154K)	42.1K (13.9K)	36.4K (9,161)	45.6K (13.8K)	26.3K (8,639)	29.9K (12.5K)	21.0K (5,526)
CD275 PE	42.6K (23.6K)	4,428 (2,037)	3,114 (1,253)	3,750 (1,193)	2,648 (1,715)	7,711 (3,581)	9,208 (7,607)	10.6K (13.4K)
CD155 PE-Dazzle594	2,267 (4,547)	4,920 (1,924)	3,348 (2,185)	5,959 (2,274)	548 (1,272)	5,686 (2,187)	4,112 (3,261)	1,857 (2,695)
CD83 PE-Cy5	10.0K (9,395)	1,150 (2,536)	-401 (3,926)	-1,216 (3,149)	1,091 (2,694)	-611 (5,420)	-246 (7,224)	-1,248 (5,628)
CCR2 PE-Cy7	121K (81.0K)	50.5K (15.3K)	170K (48.1)	297K (73.6K)	155K (48.3K)	316K (48.6K)	147K (115K)	14.7K (11.5K)
CD19 Spark NIR 685	277 (881)	-1,123 (565)	-100 (1,377)	-1,844 (1,156)	-1,980 (1,241)	-2,286 (1,415)	-1,334 (904)	-580 (471)
CD303 APC-Fire 750	72.8K (42.9K)	3,170 (1,640)	672 (1,213)	-352 (2,680)	47.2K (10.2K)	-73 (1,545)	12,119 (16,282)	11,622 (15,160)

Collectively, this indicates that HLA-DR-positive phenotypes other than B cells, monocytes, and dendritic cells are increased in patients with RA and that these phenotypes share features of granulocytes (CD15 and CD16, high side scatter) while also expressing markers associated with co-stimulation (CD275, ICOS-L) and dendritic-cell-associated molecules CD83 (18) and CD303 (20). We then focused on the potential relationship of these HLA-DR+CD15+CD16+ cells with clinical inflammation in RA.

### HLA-DR+CD15+CD16+ phenotypes correlate with clinical markers of inflammation

**Figure 5:**
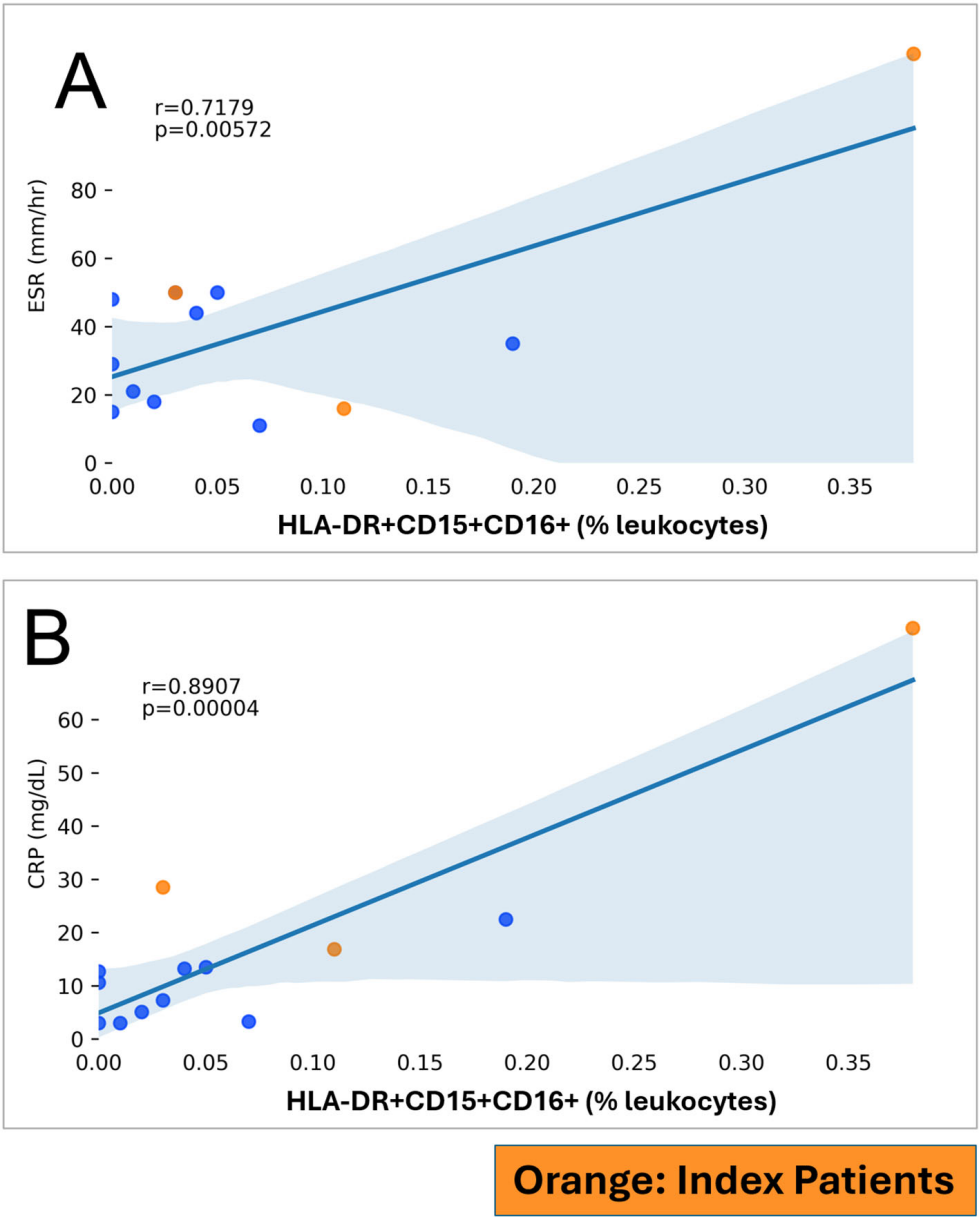
HLA-DR+CD15+CD16+ (in % leukocytes; y-axis) and markers of inflammation (x-axis) in RA patients. RA index patients are highlighted separately. **(A)** ESR, Erythrocyte sedimentation rate. **(B)** CRP, C-reactive protein.

We correlated HLA-DR+CD15+CD16+ and CD15+CD16+ phenotypes with erythrocyte sedimentation rate and serum C-reactive protein levels, commonly used clinical markers of systemic inflammatory activity (21). We saw a strong correlation between HLA-DR+CD15+CD16+ and erythrocyte sedimentation rate (*r* = 0.7179, *p* = 0.0057; **Figure 5A**) and C-reactive protein (*r* = 0.8907, *p* < 0.0001; **Figure 5B**). These correlations with clinical markers of inflammation implicate HLA-DR+CD15+CD16+ and are suggestive of their potential clinical significance in the systemic inflammatory disease RA. CD15+CD16+ was correlated with erythrocyte sedimentation rate (*r* = 0.5866, *p* = 0.0351; **Supplementary Figure S22A**) but not with C-reactive protein (*p* = 0.1403; **Supplementary Figure S22B**).

## Discussion

The striking HLA-DR (MHC II) association with RA (3, 4) and the fact that MHC II molecules are involved in capturing and processing antigens point toward an important role of HLA-DR+ monocytes and dendritic cells in RA which is supported by existing characterizations (22–28). Here we used HLA-DR as a broader APC definition to capture and comprehensively characterize dendritic cells and monocytes as well as other potential antigen-presenting cells using spectral cytometry. We used the t-SNE screens of index patients to develop working definitions which we then expanded to a larger patient cohort for confirmation.

We found that RA antigen-presenting cells have raised CD56 in DC2 and DC3 and intermediate monocytes and increased the surface expression of the co-stimulatory molecules CD86 and CD275 (ICOS-L) in DC2. CD56 is commonly used to identify NK cells. Alternatively, CD56 can be expressed by other cell types, including antigen-presenting cells (7), which is why we retained it for this study. Since, on the other hand, HLA-DR has been observed to be inducible on NK cells (15), it raised the initial question on whether CD56 increases in dendritic cells that we observed could be explained by the acquisition of HLA-DR and other dendritic cell/monocyte markers by NK cells. We found this to be unlikely: CD56+ NK cells consistently formed distinct populations in CD56/HLA-DR plots—making them easily distinguishable on contour plots (**Figure 2**). The HLA-DR expression of these NK cells was far below the HLA-DR+ gating threshold. Consistently, unsupervised SPADE classifications indicated that NK cells were not present in the APC gate (**Supplementary Figures S1G, S2G**). CD56 can function as a molecule for homophilic adhesion (7), which raises the possibility that the CD56 increases in antigen-presenting cells increase the likelihood and intensity of their interactions with CD56+ NK cells. In a similar vein, RA patients had a widespread increase of CD15 across monocyte and dendritic cell populations (with the exception of non-classical monocytes). CD15, a post-translationally modified glycan determinant, mediates adhesion between myeloid cells (13). In contrast, broad increases of HLA-DR—to suggest increased antigen presentation—were not seen in this study. Except for RA B lymphocytes (where HLA-DR was indeed increased, as anticipated), HLA-DR was not increased in RA monocytes and dendritic cells.

In combination, our data suggest that the participation of myeloid APC in RA is much more nuanced than simply a global increase of antigen presentation through HLA-DR. Indeed highly inflammatory severe systemic inflammatory states have been shown to be associated with decreased HLA-DR (29, 30). Our findings instead implicate the increased adhesive capacity of APC (CD15 and CD56 increases) and enhanced co-stimulation.

Beyond changes in dendritic cells and monocytes, we found that HLA-DR+CD15+CD16+ cells contribute to the HLA-DR+ APC compartment in RA. In the context of the widely increased CD15 pointing toward altered myelopoiesis, their existence may be a related phenomenon. Their HLA-DR+ CD83+ co-stimulatory (CD275+) phenotype suggests that these cells may be capable to provide co-stimulatory signals to CD4+ T cells and may thus participate in adaptive immunity by exerting a proinflammatory influence on T cells. One possibility is to conceptualize them as a subset of activated low-density granulocytes (LDG), which were originally described as a “contaminant” found in rheumatic diseases (31) but have since been characterized in lupus and other inflammatory conditions (32, 33). On one hand, in the RA context, our findings of (HLA-DR+)CD15+CD16+ cells align with a previous report and characterization of RA LDG which found RA LDG to be distinct from RA neutrophils and to have impaired TNF signaling (34). On the other hand, unlike what is known about LDG, they expressed CD303, associated with plasmacytoid pDC (20) and CD83 and CD275 (ICOS-L), that are associated with antigen-presenting cells (18, 19). Given their phenotypic overlap between granulocytes and monocytes/dendritic cells, they are, in our view, similarly reminiscent of a reported neutrophil-DC “hybrid” cell concept (35). Overlapping HLA-DR+ phenotypes of neutrophils and dendritic cells have also been implicated in multiple sclerosis (36) and leishmaniasis (37).

**Figure 6:**
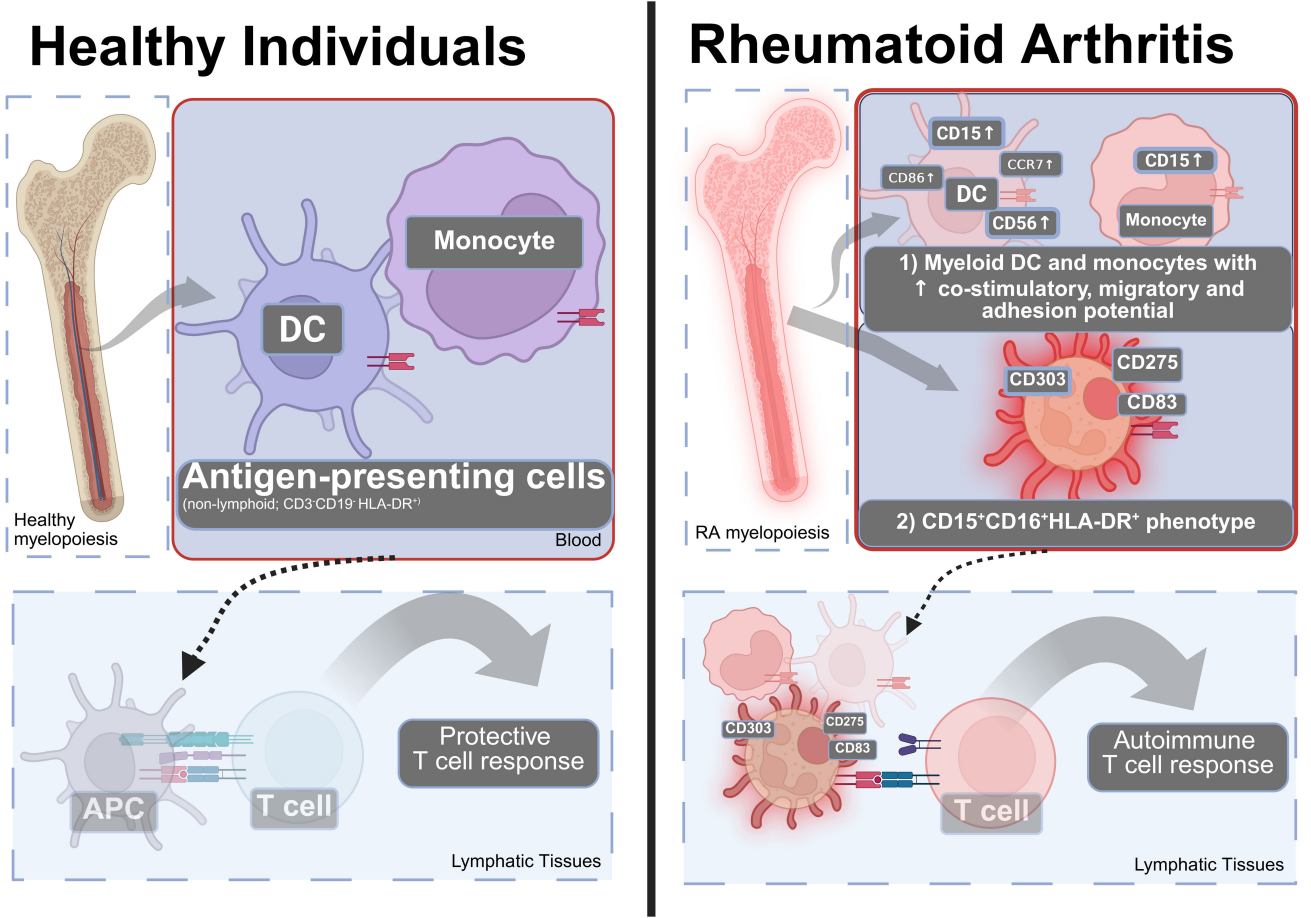
A model of how the changes in the blood APC (HLA-DR+) compartment can contribute to the pathophysiology of RA. Left: In healthy individuals, the blood APC compartment contains DC and monocytes that can present antigen to induce protective T cell responses. Right: In RA, there is (1) an increase in co-stimulatory molecules, adhesion and migratory molecules in dendritic cells, and monocytes and (2) appearance of an CD15+CD16+HLA-DR+ phenotype that is positive for CD83, CD275—consistent with pro-inflammatory co-stimulation, migration, and adhesive properties suggesting involvement by altered antigen presentation to T lymphocytes. Many additional cell types contribute to RA. Created using Biorender.

Overall, our findings suggest that RA patients have altered myeloid cells that are characterized by increased adhesive, migratory, and co-stimulatory potential in multiple lineages without directly affecting their HLA-DR expression (**Figure 6**). These observations point toward fundamental alterations of myeloid/bone marrow cells with the important caveat that the direct experimental confirmation of these potential mechanisms was beyond the scope of this immunophenotyping study.

The strengths of our study include a comprehensive immunophenotyping perspective of monocytes and dendritic cells that retains all antigen-presenting cells (HLA-DR+) for analysis. We focused on patients with clinically “striking” disease activity to create working definitions and confirmed our observations in a larger patient cohort. The study limitations include sample size and focus on the peripheral blood. Despite the indication of an extensive CD56 increase in monocytes in the initial t-SNE screens, we did not observe statistically significant increases of CD56 in monocytes, potentially due to the limited sample size. We used HLA-DR positivity only as a screening criterion for APC; HLA-typing was not within the scope of the study. Clinical disease activity scores were not available. Additional alternative dendritic cell definitions exist—for example, DC1 can be distinguished using CLEC9A (38). During panel development, we noticed an almost perfect co-expression of CD141 with CLEC9A on DC1. For this reason, CLEC9A was not included and DC1 were gated using CD141.

Another future question is the response of APC phenotypes to different RA treatments. In our index patients, normalization of the CD15+CD16+ and HLA-DR+CD15+CD16+ populations was attributed to treatment which included methotrexate, raising the possibility that adenosine signaling (39) may be beneficial and underlie the improvement seen in these patients.

This high-level HLA-DR-centric overview suggests that multiple, overlapping myeloid lineages are affected through altered adhesive (CD15 and CD56) and increased co-stimulatory capacity (CD86, CD275 (ICOS-L)) in RA. These myeloid changes could explain aspects of autoimmune diseases and represent potential new research and therapeutic targets, particularly for RA manifestations that are difficult to treat with existing therapies. We hope that our study will encourage the comprehensive perspective on dendritic cells, monocytes, and alternative APC phenotypes in blood and other tissues to help inform new rational therapies for RA patients.

## Data Availability

The datasets presented in this study can be found in online repositories. The names of the repository/repositories and accession number(s) can be found in the article/**Supplementary Material**.
