# Supplementary material for: Spectral cytometry of rheumatoid arthritis patients implicates myeloid dendritic cells and granular HLA-DR+CD15+CD16+ cells in pro-inflammatory antigen presentation
Source: Front Immunol. 2025 Jul 9;16:1596609. doi: 10.3389/fimmu.2025.1596609 (PMC12283323; doi:10.3389/fimmu.2025.1596609)
Supplement: Supplementary file 1 [file DataSheet1.docx]

Supplementary Material

## Supplementary Figures


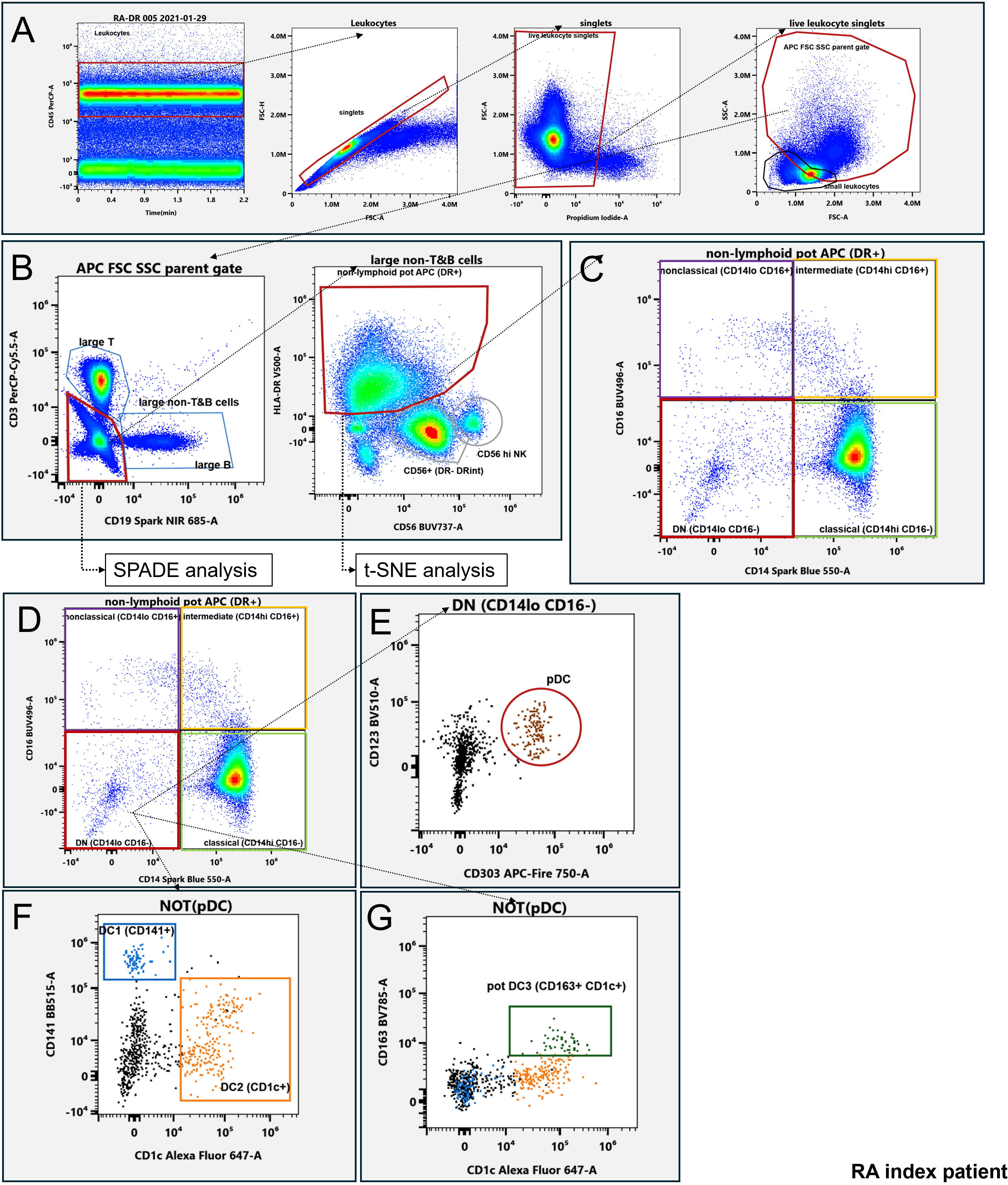


**Supplementary Figure 1**. Gating strategy for monocytes and dendritic cells – representative RA index patient. We gated large live singlet CD45+ leukocytes (A). Lymphoid cells (CD3+, CD19+) and CD56+ NK cells and other HLA-DRneg/lo cells were gated out. Cells contained in the ‘non-lymphoid pot APC (DR+)’ gates (B, red gate) were used for additional downstream t-SNE analyses (B) Classical (green), intermediate (yellow), and non-classical monocytes (purple) were gated by CD14 and CD16 expression (C, D) Plasmacytoid pDC were gated as CD123+CD303+ (E) DC1 were gated as CD141+ (blue) and DC2 as CD1c+ (orange) (F). DC3 were gated as CD163+CD1c+ (G)


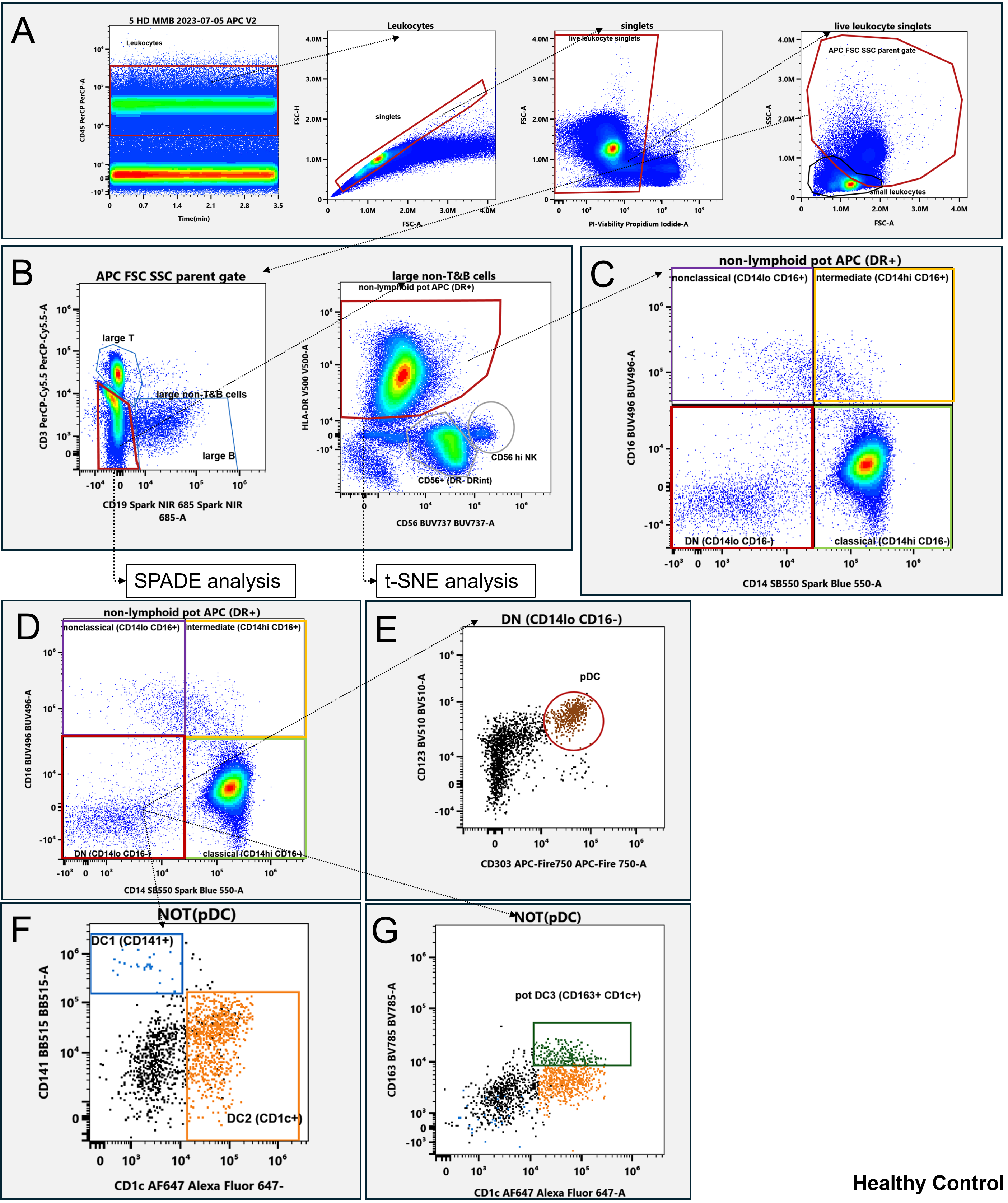


**Supplementary Figure 2**. Gating strategy for monocytes and dendritic cells – representative healthy control donor. We gated large live singlet CD45+ leukocytes (A). Lymphoid cells (CD3+, CD19+) and CD56+ NK cells and other HLA-DRneg/lo cells were gated out. Cells contained in the ‘non-lymphoid pot APC (DR+)’ gates (B, red gate) were used for additional downstream t-SNE analyses (B) Classical (green), intermediate (yellow), and non-classical monocytes (purple) were gated by CD14 and CD16 expression (C, D) Plasmacytoid pDC were gated as CD123+CD303+ (E) DC1 were gated as CD141+ (blue) and DC2 as CD1c+ (orange) (F). DC3 were gated as CD163+CD1c+ (G)


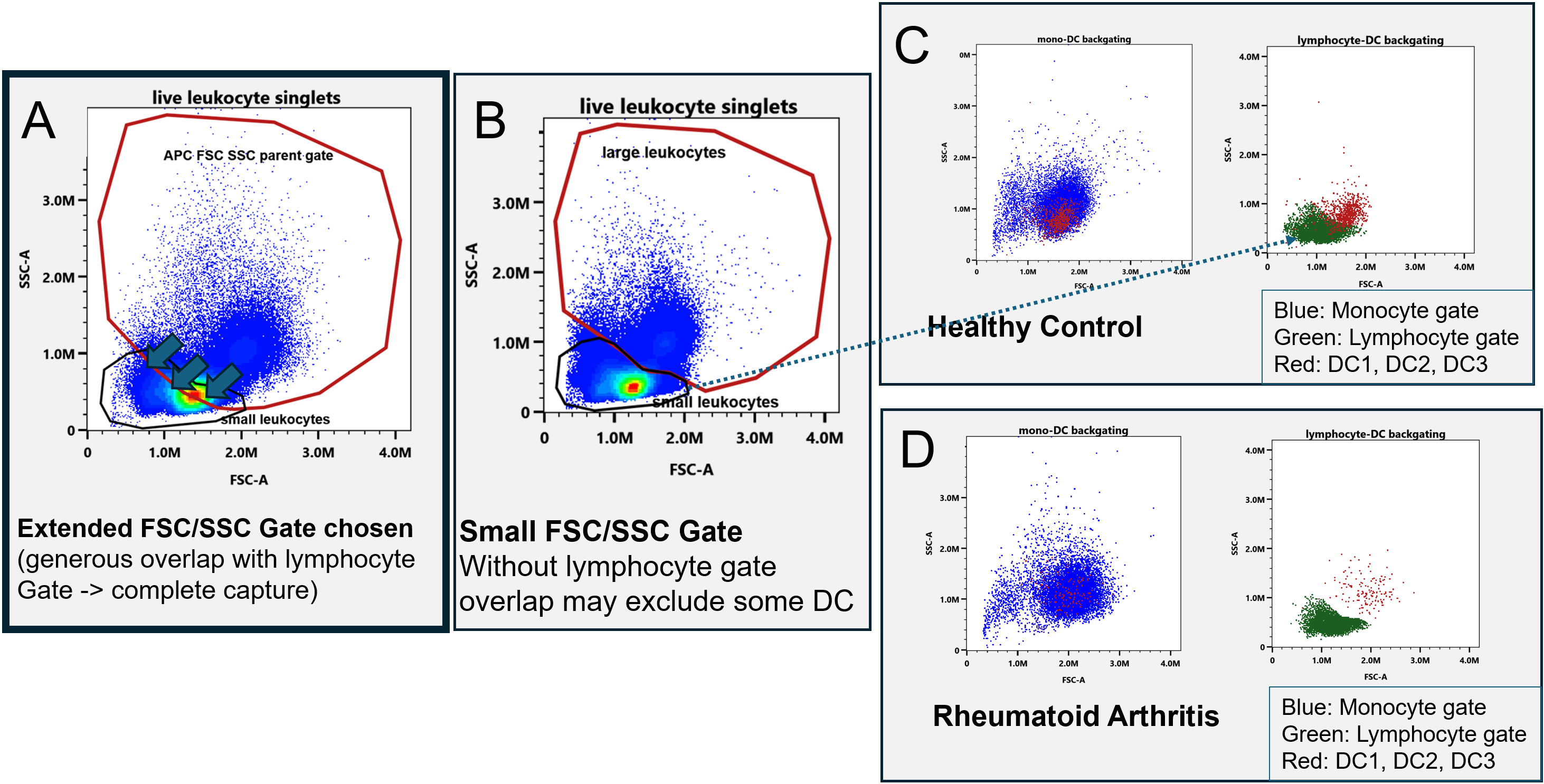


**Supplementary Figure 3**. Determining size and morphology of the FSC/SSC gate to optimize APC capture. Generous FSC/SSC gate (A). Small FSC/SSC gate (B). Representative examples for healthy control (C) and RA (D). Viable dendritic cells (red), monocytes (blue), and lymphocytes (green) were first gated based on surface marker expression alone and then back-gated to inform the optimal FSC/SSC gate that ensures the complete capture of DC and monocytes. (healthy control (C) and RA patient (D) confirmed significant overlap with lymphocyte populations particularly in healthy controls (C, red and green populations). For this reason, an extended FSC/SSC gate, seen in A, that ensures the capture of all DC, was used. DC: dendritic cells FSC: forward scatter SSC: side scatter


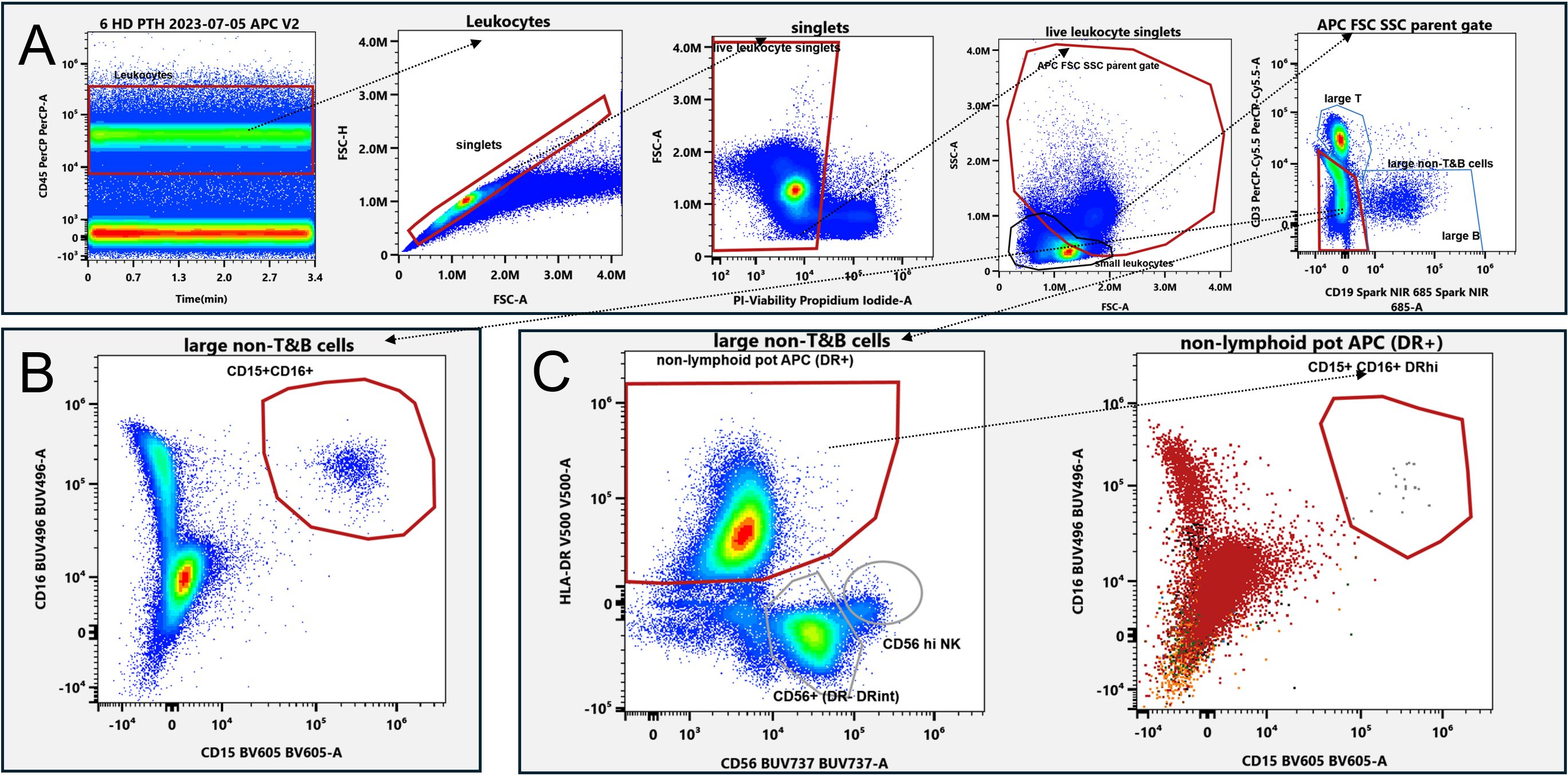


**Supplementary Figure 4**. Gating strategy for CD15+CD16+ and HLA-DR+CD15+CD16+ cells. We gated live singlet CD45+ leukocytes (A). The FSC/SSC gate was set generously to ensure full capture of granulocytic populations (fourth plot, red gate, also see Supplementary Figure 3). Lymphoid cells (fifth plot, CD3+, CD19+) were gated out. (B) Non-lymphoid HLA-DR+ population was gated (red gate) (C) HLA-DR+CD15+CD16+ cells were gated (red gates); CD56+ NK cells and other HLA-DR negative cells were gated out. Healthy control donor shown.


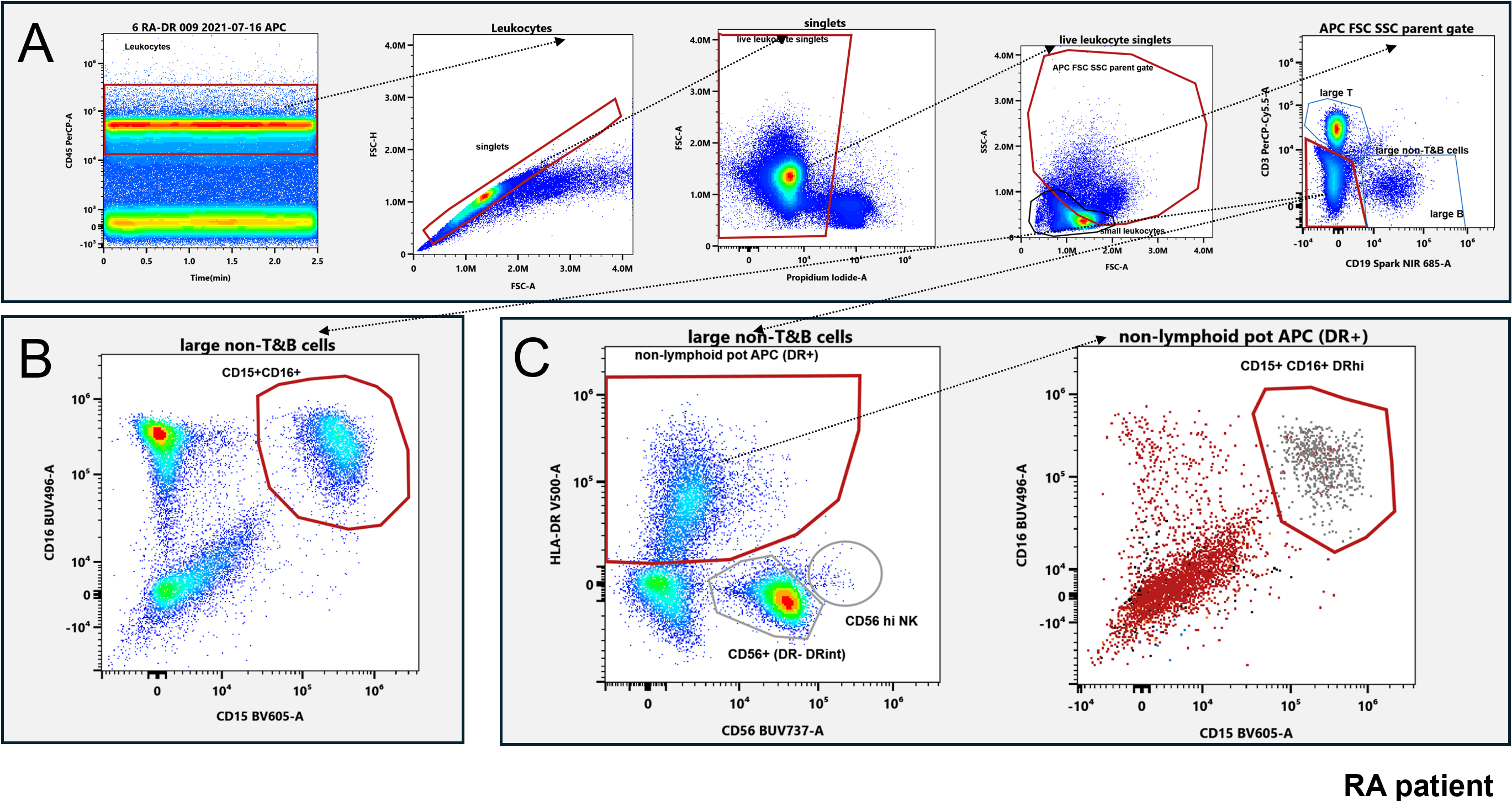


**Supplementary Figure 5**. Gating strategy for CD15+CD16+ and HLA-DR+CD15+CD16+ cells. (A) We gated live singlet CD45+ leukocytes. The FSC/SSC gate was widened to ensure full capture of granulocytic populations (fourth plot, red gate). Lymphoid cells (fifth plot, CD3+, CD19+) were gated out. (B) CD15+CD16+ population was gated (red gate) (C) HLA-DR+CD15+CD16+ cells were gated (red gates); CD56+ NK cells and other HLA-DR negative cells were gated out. RA index patient with polyarticular synovitis shown.


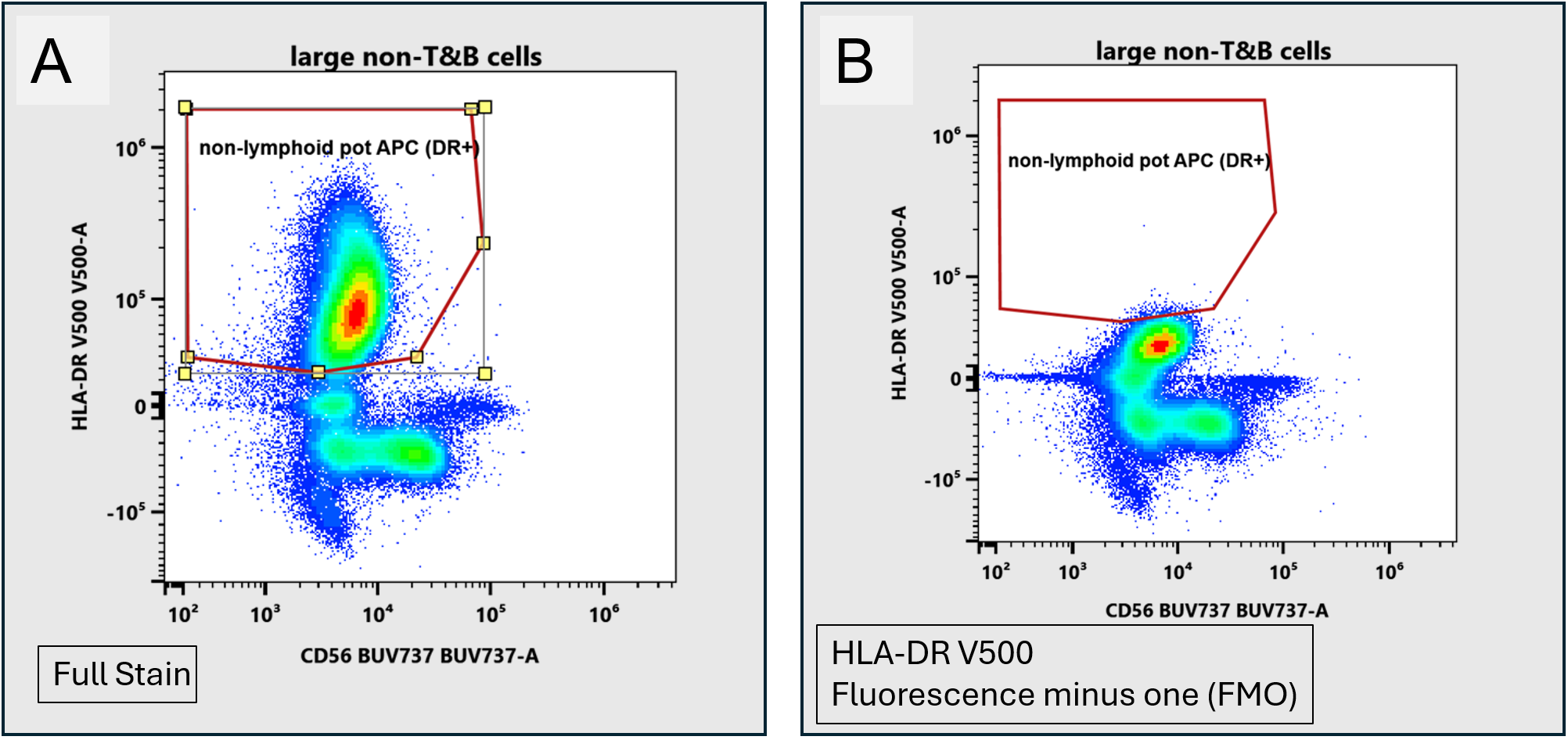


**Supplementary Figure 6**. Fluorescence minus one (FMO) for HLA-DR (A) Peripheral blood mononuclear cells (PBMC) from a healthy donor were stained with full panel reagents listed in Table 2. (B) PBMCs from the same donor were stained with the full panel but lacking HLA-DR V500. Gated on live CD3-CD19- single cells.


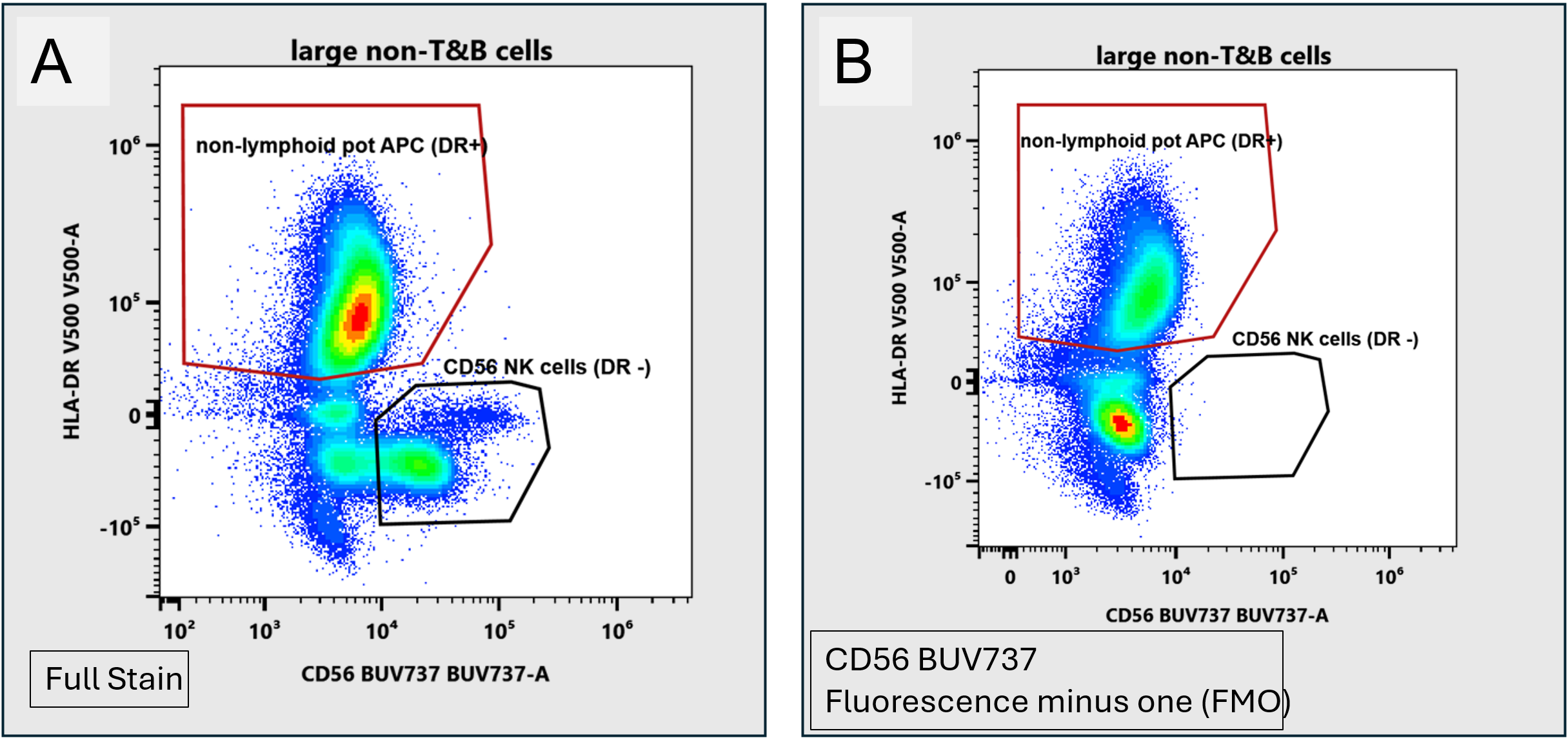


**Supplementary Figure 7**. Fluorescence minus one (FMO) for CD56 (A) Peripheral blood mononuclear cells (PBMC) from a healthy donor were stained with full panel reagents listed in Table 2. (B) PBMCs from the same donor were stained with the full panel but lacking CD56 BUV737. Gated on live CD3-CD19- single cells.


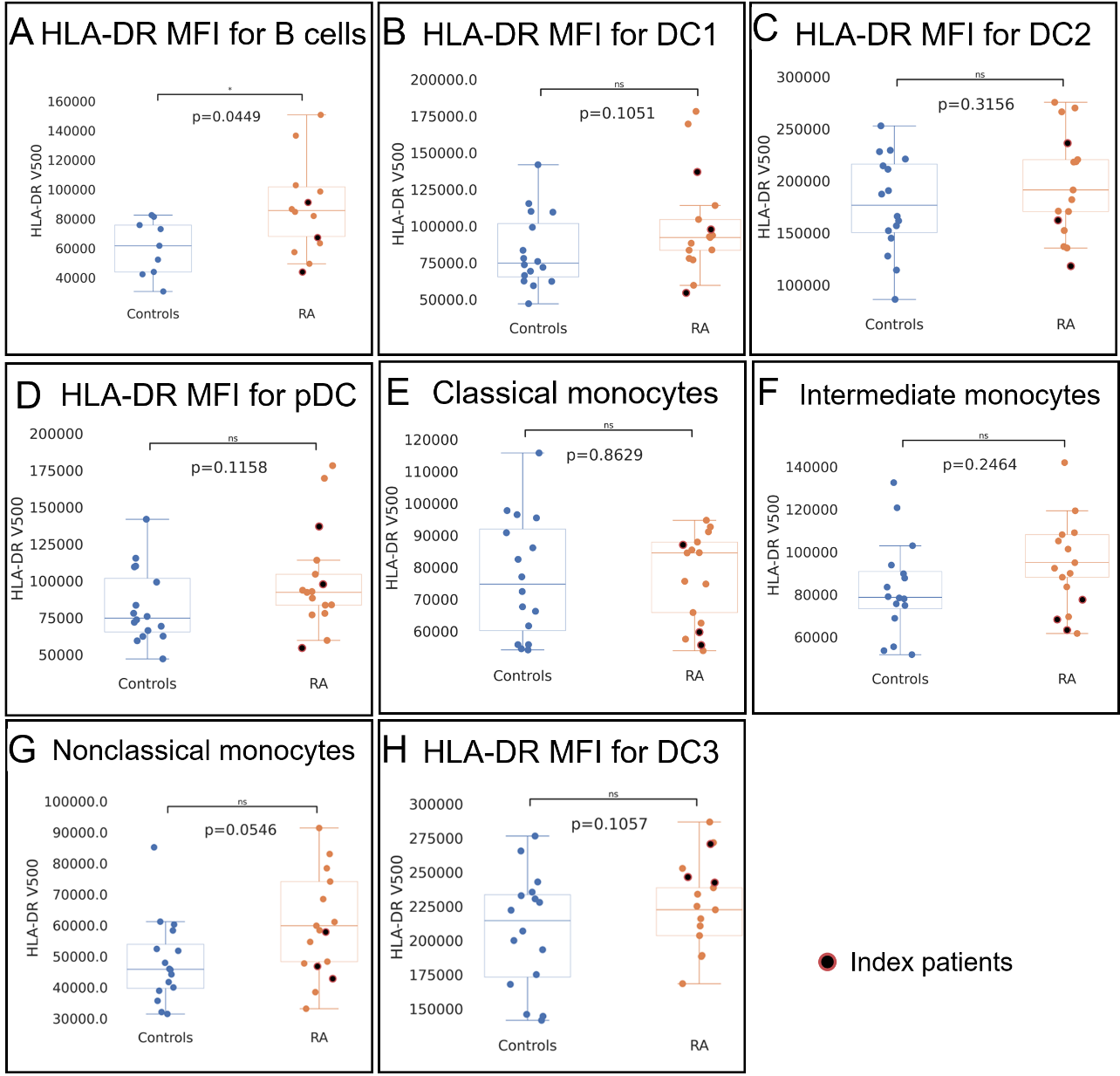


**Supplementary Figure 8**. HLA-DR in antigen-presenting cells, measured by spectral flow cytometry. (A) B cells (B) DC1 (CD141+ dendritic cells) (C) DC2 (CD1c+ dendritic cells) (D) CD123+CD303+ plasmacytoid dendritic cells (E) classical monocytes (CD14hiCD16lo) (F) intermediate monocytes (CD14hiCD16+) (G) nonclassical monocytes (CD14-CD16hi) (H) DC3 (CD1c+CD163+ dendritic cells). Blue: healthy control donors. Orange: RA. RA index patients are highlighted in red.


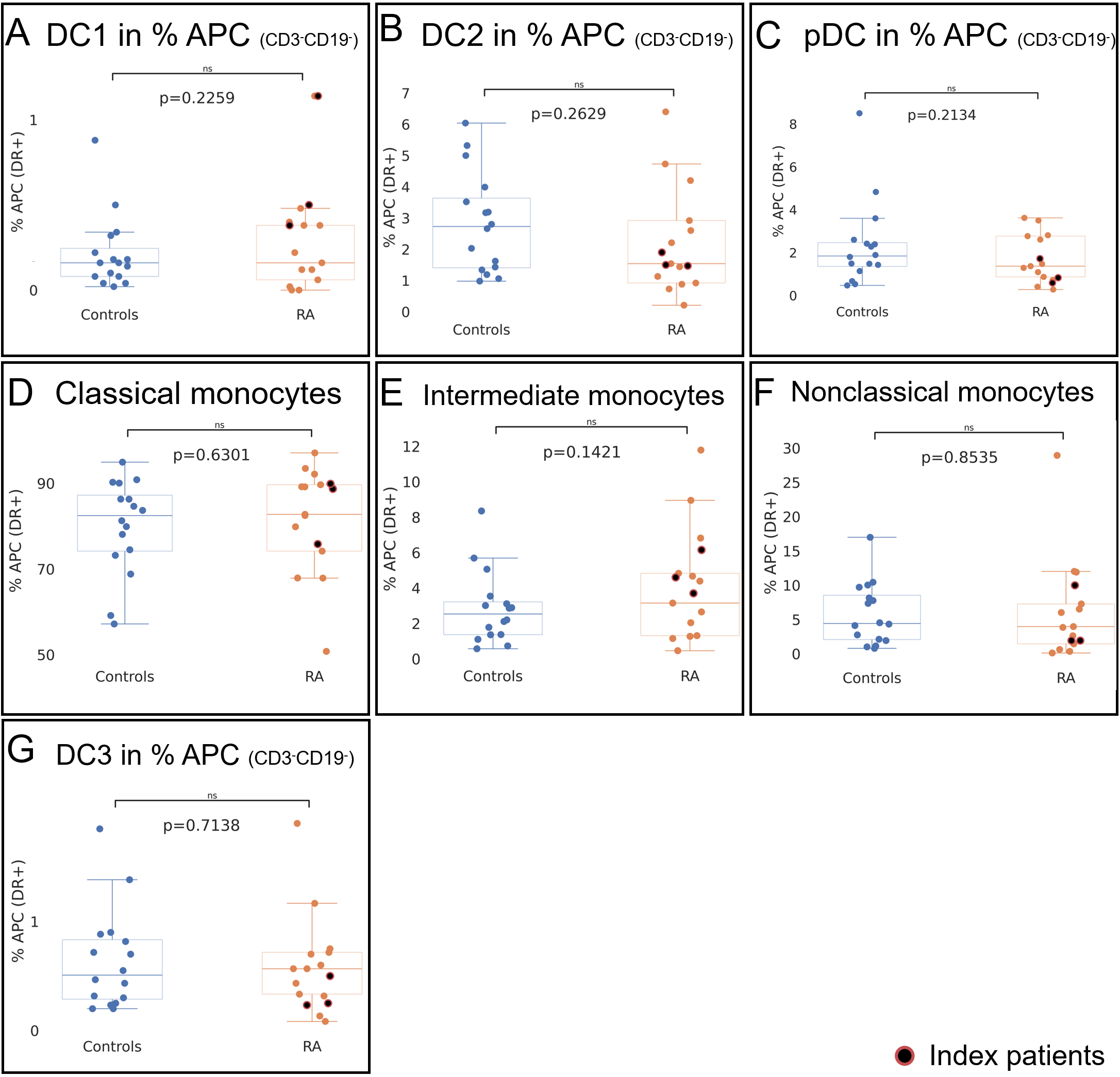


**Supplementary Figure 9**. Antigen-presenting cell subsets (HLA-DR+), measured by spectral flow cytometry as percentages of non-lymphoid APC (HLA-DR+CD3-CD19-). (A) DC1 (CD141+ dendritic cells) (B) DC2 (CD1c+ dendritic cells) (C) CD123+CD303+ plasmacytoid dendritic cells (D) classical monocytes (CD14hiCD16lo) (E) intermediate monocytes (CD14hiCD16+) (F) nonclassical monocytes (CD14-CD16hi) (G) DC3 (CD1c+CD163+ dendritic cells). Blue: healthy control donors. Orange: RA. RA index patients are highlighted in red.


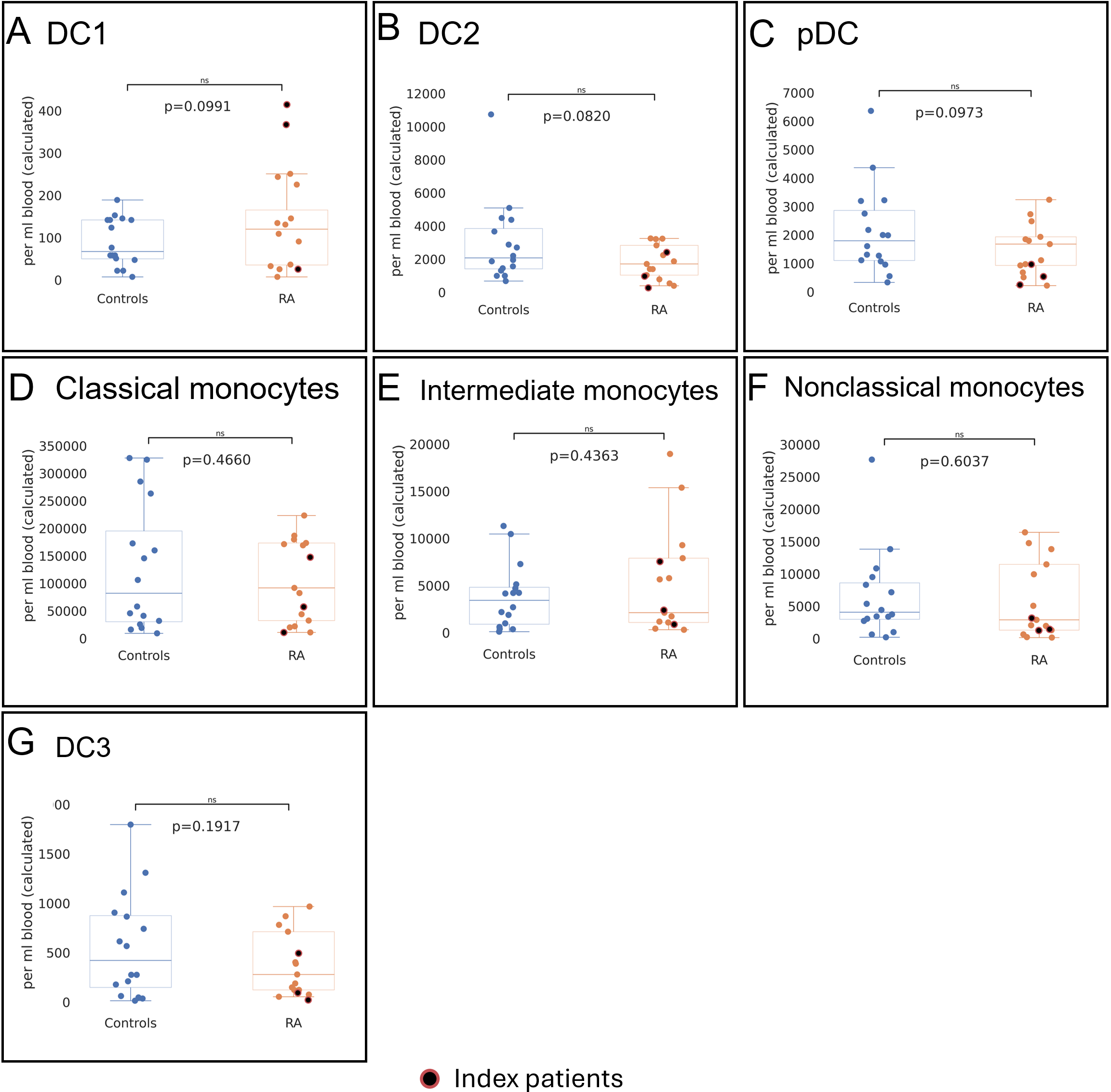


**Supplementary Figure 10**. Antigen-presenting cell subsets (HLA-DR+), measured by spectral flow cytometry per ml of blood. (A) DC1 (CD141+ dendritic cells) (B) DC2 (CD1c+ dendritic cells) (C) CD123+CD303+ plasmacytoid dendritic cells (D) classical monocytes (CD14hiCD16lo) (E) intermediate monocytes (CD14hiCD16+) (F) nonclassical monocytes (CD14-CD16hi) (G) DC3 (CD1c+CD163+ dendritic cells). Blue: healthy control donors. Cell numbers per ml of blood were calculated by dividing cell counts by the sample volume, then multiplied by the volume of blood used to prepare each analyzed sample. Orange: RA. RA index patients are highlighted in red.


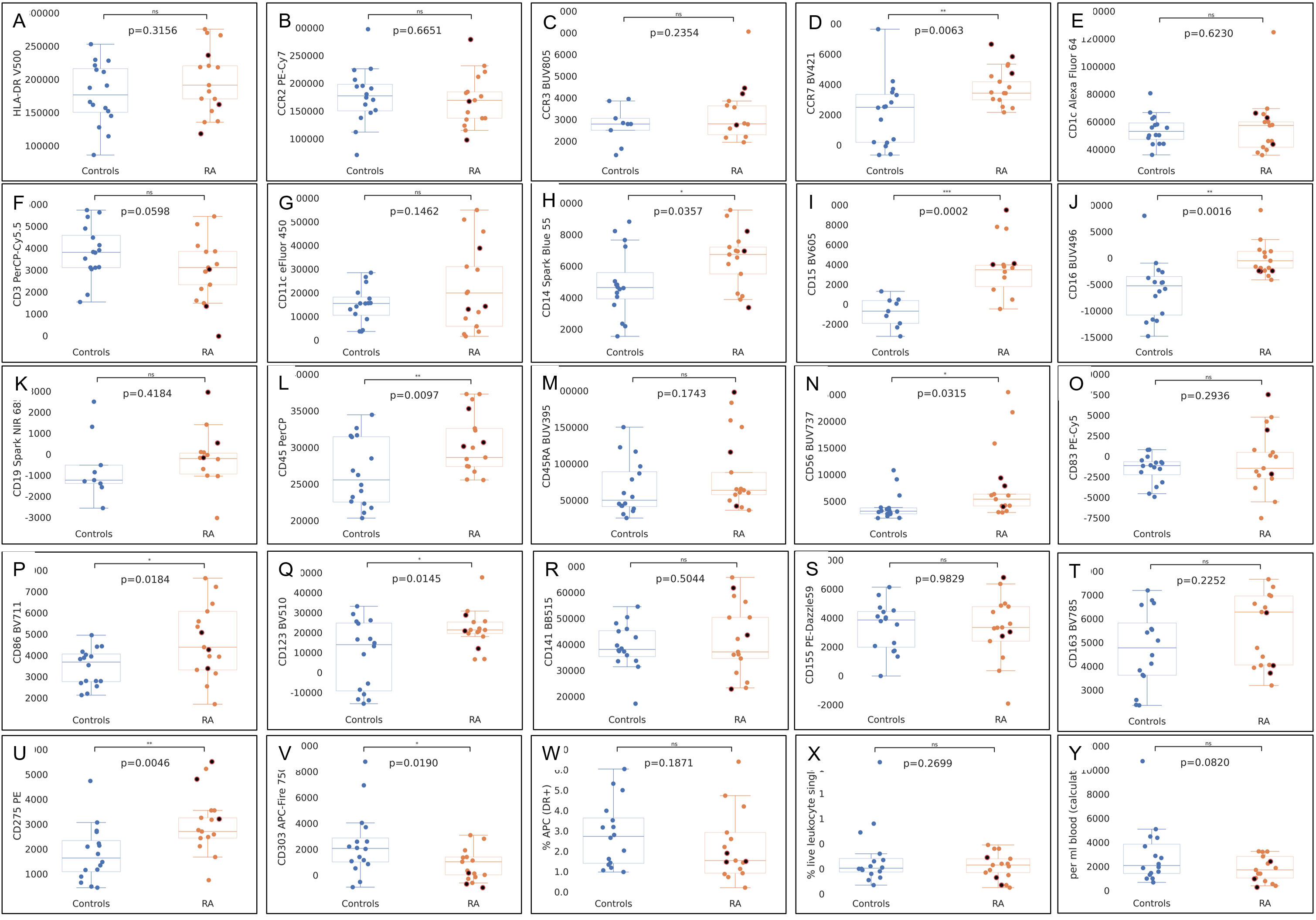


**Supplementary Figure 11**. Immunophenotyping results of CD1c+ dendritic cells (DC2) of RA (orange) and healthy control donors (blue). (A) HLA-DR V500 (B) CCR2 PE-Cy7 (C) CCR3 BUV 805 (D) CCR7 BV421 (E) CD1c AF647 (F) CD3 PerCP-Cy5.5 (G) CD11c eFluor450 (H) CD14 SB550 (I) CD15 BV605 (J) CD16 BUV496 (K) CD19 Spark NIR 685 (L) CD45 PerCP (M) CD45RA BUV395 (N) CD56 BUV737 (O) CD83 PE-Cy5 (P) CD86 BV711 (Q) CD123 BV510 (R) CD141 BB515 (S) CD155 PE-Dazzle594 (T) CD163 BV785 (U) CD275 PE (V) CD303 APC-Fire 750. two-sided t-tests. * p<0.05 ** p<0.01 ***p<0.001


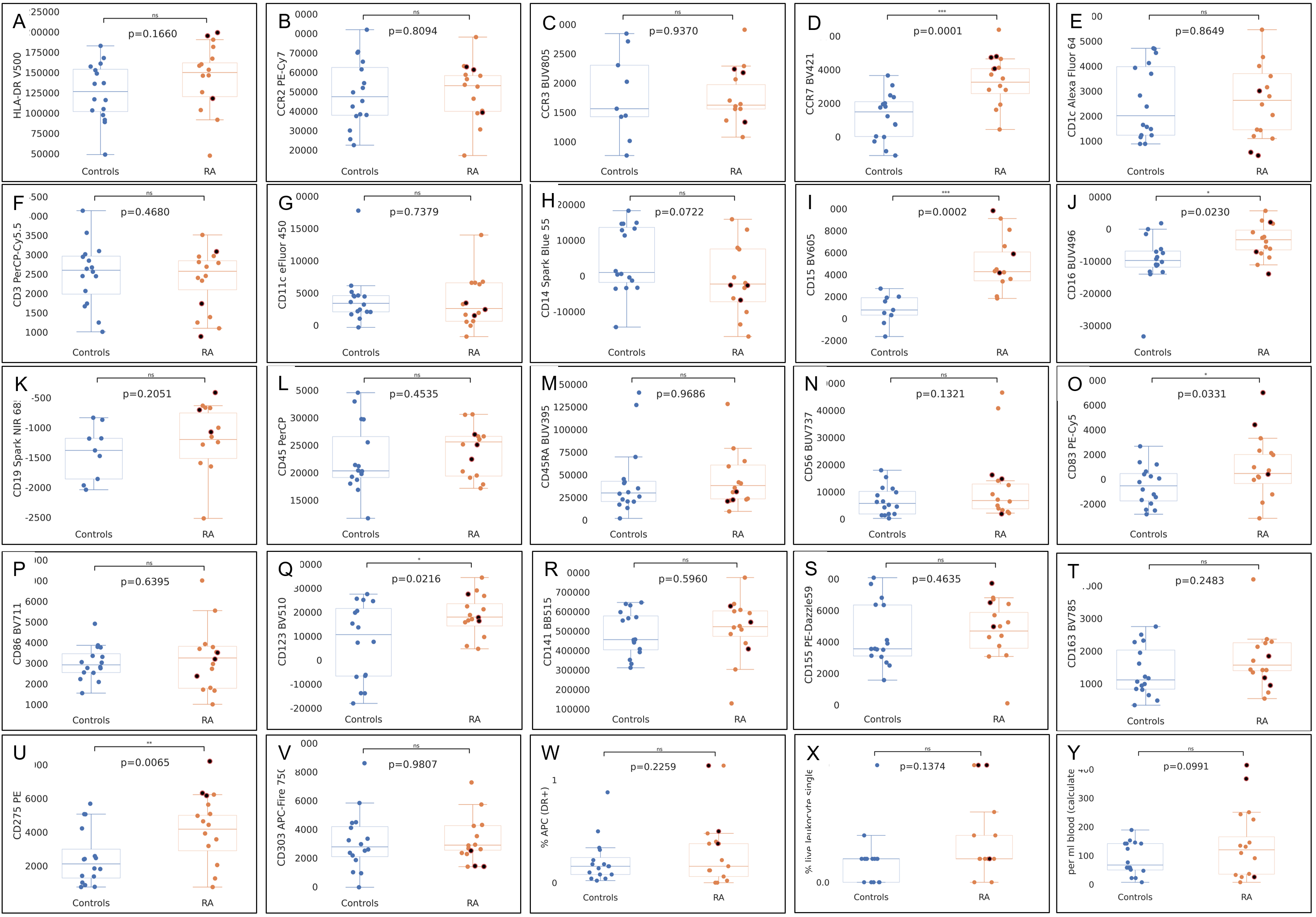


**Supplementary Figure 12**. Immunophenotyping results of CD141+ dendritic cells (DC1) of RA (orange) and healthy control donors (blue). (A) HLA-DR V500 (B) CCR2 PE-Cy7 (C) CCR3 BUV 805 (D) CCR7 BV421 (E) CD1c AF647 (F) CD3 PerCP-Cy5.5 (G) CD11c eFluor450 (H) CD14 SB550 (I) CD15 BV605 (J) CD16 BUV496 (K) CD19 Spark NIR 685 (L) CD45 PerCP (M) CD45RA BUV395 (N) CD56 BUV737 (O) CD83 PE-Cy5 (P) CD86 BV711 (Q) CD123 BV510 (R) CD141 BB515 (S) CD155 PE-Dazzle594 (T) CD163 BV785 (U) CD275 PE (V) CD303 APC-Fire 750. two-sided t-tests. * p<0.05 ** p<0.01 ***p<0.001


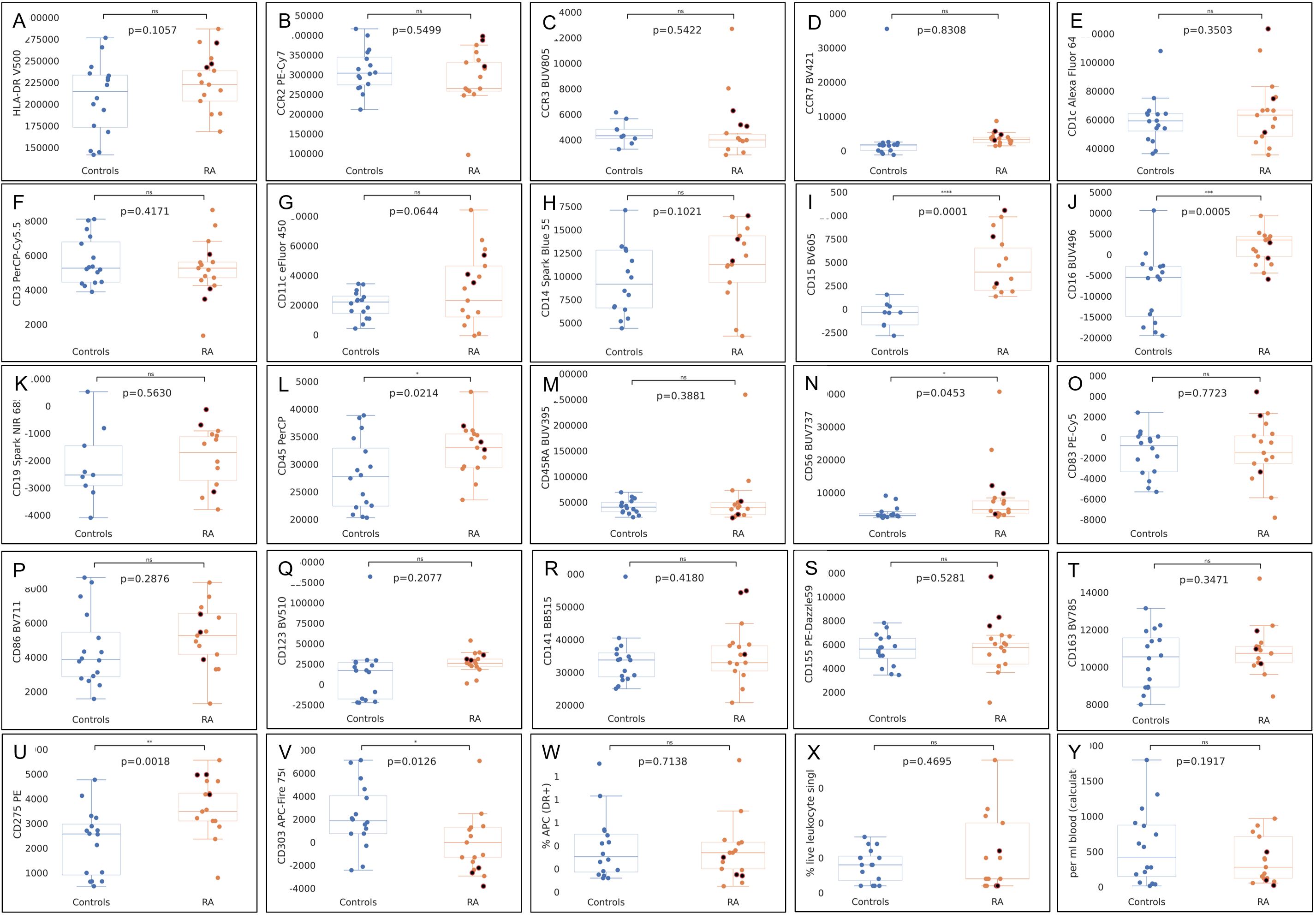


**Supplementary Figure 13**. Immunophenotyping results of CD163+CD1c+ dendritic cells (DC3) of RA (orange) and healthy control donors (blue). (A) HLA-DR V500 (B) CCR2 PE-Cy7 (C) CCR3 BUV 805 (D) CCR7 BV421 (E) CD1c AF647 (F) CD3 PerCP-Cy5.5 (G) CD11c eFluor450 (H) CD14 SB550 (I) CD15 BV605 (J) CD16 BUV496 (K) CD19 Spark NIR 685 (L) CD45 PerCP (M) CD45RA BUV395 (N) CD56 BUV737 (O) CD83 PE-Cy5 (P) CD86 BV711 (Q) CD123 BV510 (R) CD141 BB515 (S) CD155 PE-Dazzle594 (T) CD163 BV785 (U) CD275 PE (V) CD303 APC-Fire 750. two-sided t-tests. * p<0.05 ** p<0.01 ***p<0.001


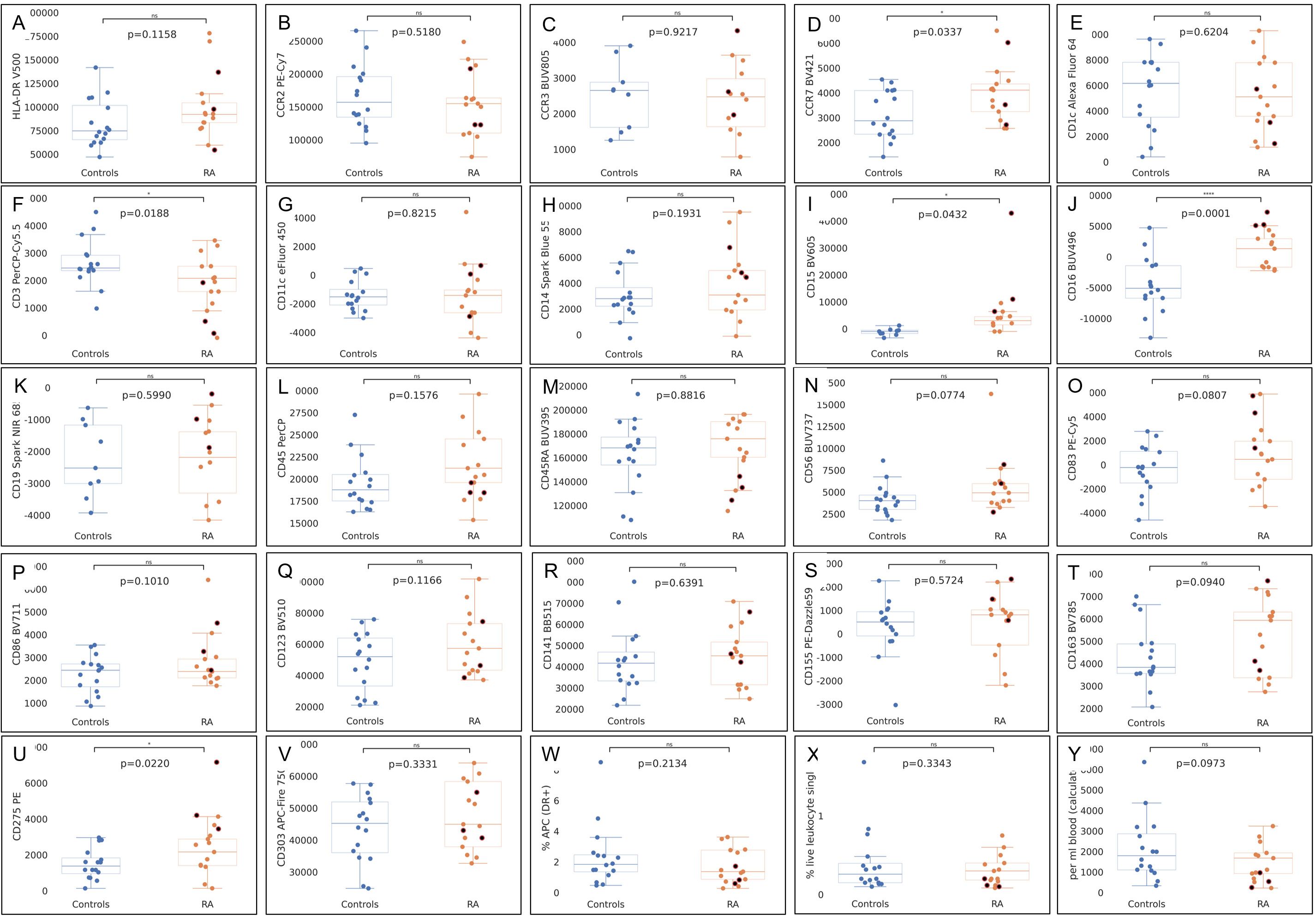


**Supplementary Figure 14**. Immunophenotyping results of plasmacytoid dendritic cells (CD123+CD303+ pDC) of RA (orange) and healthy control donors (blue). (A) HLA-DR V500 (B) CCR2 PE-Cy7 (C) CCR3 BUV 805 (D) CCR7 BV421 (E) CD1c AF647 (F) CD3 PerCP-Cy5.5 (G) CD11c eFluor450 (H) CD14 SB550 (I) CD15 BV605 (J) CD16 BUV496 (K) CD19 Spark NIR 685 (L) CD45 PerCP (M) CD45RA BUV395 (N) CD56 BUV737 (O) CD83 PE-Cy5 (P) CD86 BV711 (Q) CD123 BV510 (R) CD141 BB515 (S) CD155 PE-Dazzle594 (T) CD163 BV785 (U) CD275 PE (V) CD303 APC-Fire 750. two-sided t-tests. * p<0.05 ** p<0.01 ***p<0.001


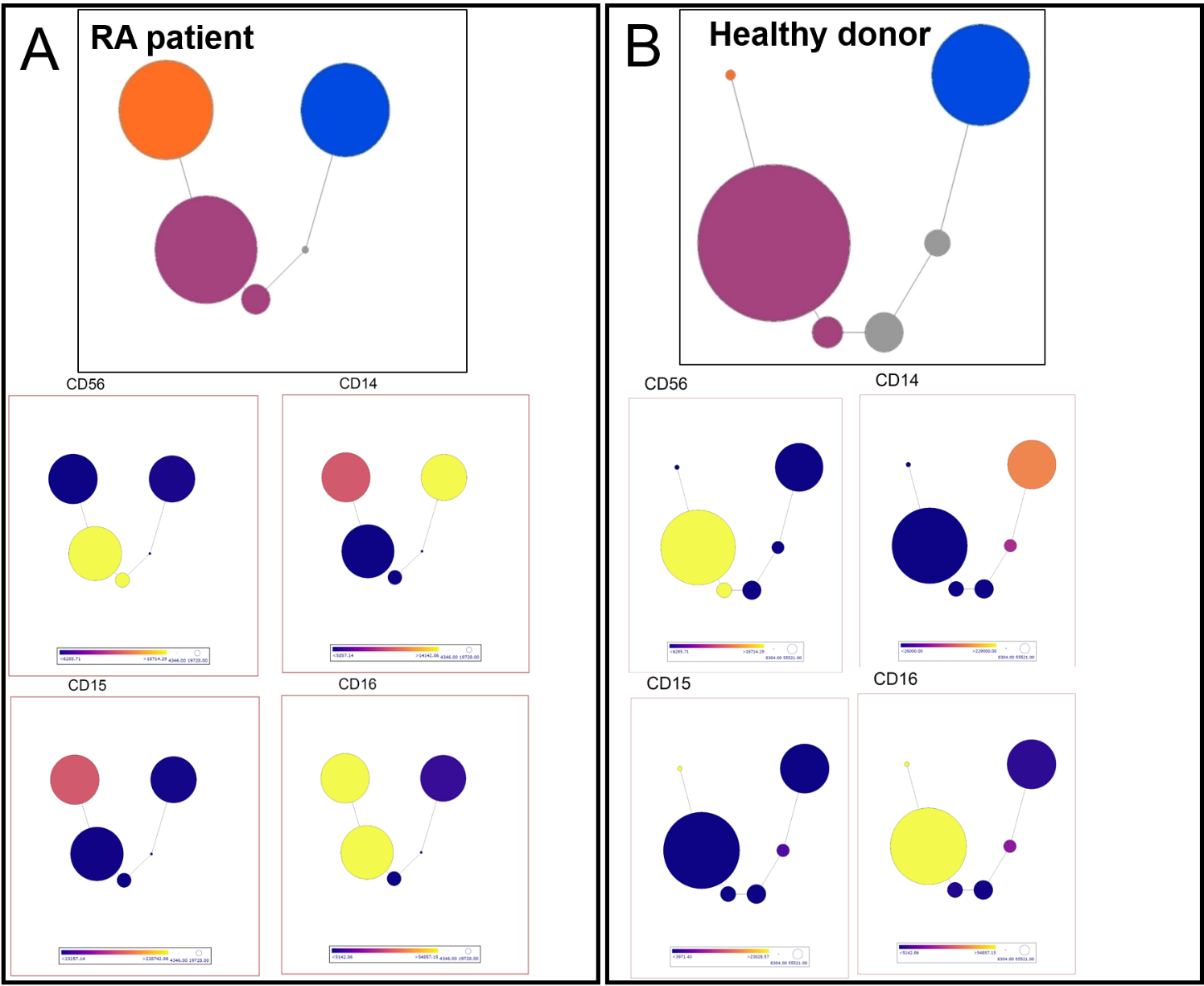


**Supplementary Figure 15.** SPADE clustering of non-lymphoid (CD3-CD19-) peripheral blood mononuclear cells. NK and monocyte clustering results were back-gated in bi-axial view in Figure 3. (A) RA patient (B) Healthy control donor. Blue: monocytes (CD56+) Purple: NK cells Orange: CD15+ low density granulocytes. Gray cluster represent a combination of pDC and DC1 (CD141+) and DC2 (CD1c+).


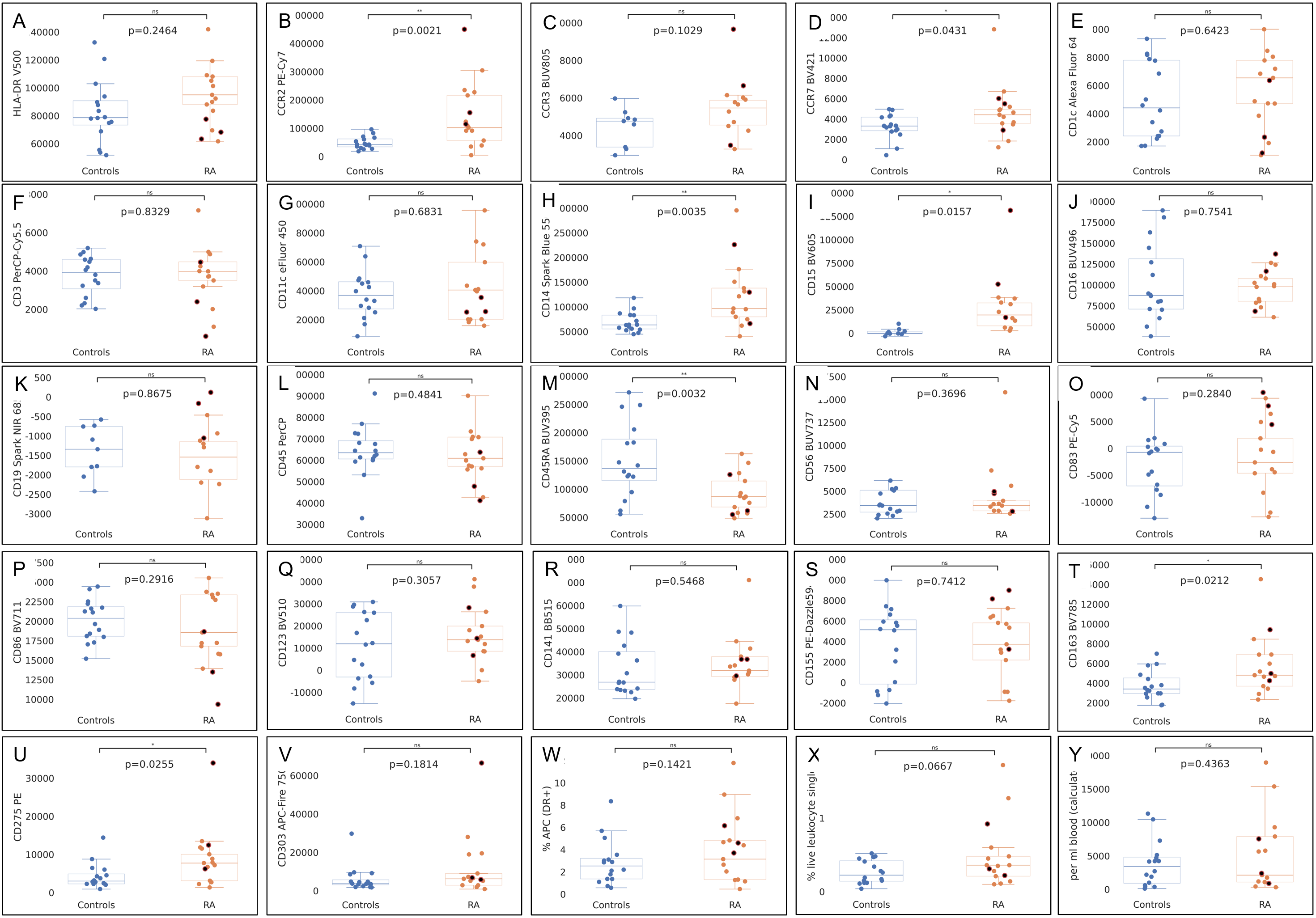


**Supplementary Figure 16**. Immunophenotyping results of CD14hiCD16+ monocytes (intermediate) of RA (orange) and healthy control donors (blue). (A) HLA-DR V500 (B) CCR2 PE-Cy7 (C) CCR3 BUV 805 (D) CCR7 BV421 (E) CD1c AF647 (F) CD3 PerCP-Cy5.5 (G) CD11c eFluor450 (H) CD14 SB550 (I) CD15 BV605 (J) CD16 BUV496 (K) CD19 Spark NIR 685 (L) CD45 PerCP (M) CD45RA BUV395 (N) CD56 BUV737 (O) CD83 PE-Cy5 (P) CD86 BV711 (Q) CD123 BV510 (R) CD141 BB515 (S) CD155 PE-Dazzle594 (T) CD163 BV785 (U) CD275 PE (V) CD303 APC-Fire 750. two-sided t-tests. * p<0.05 ** p<0.01 ***p<0.001


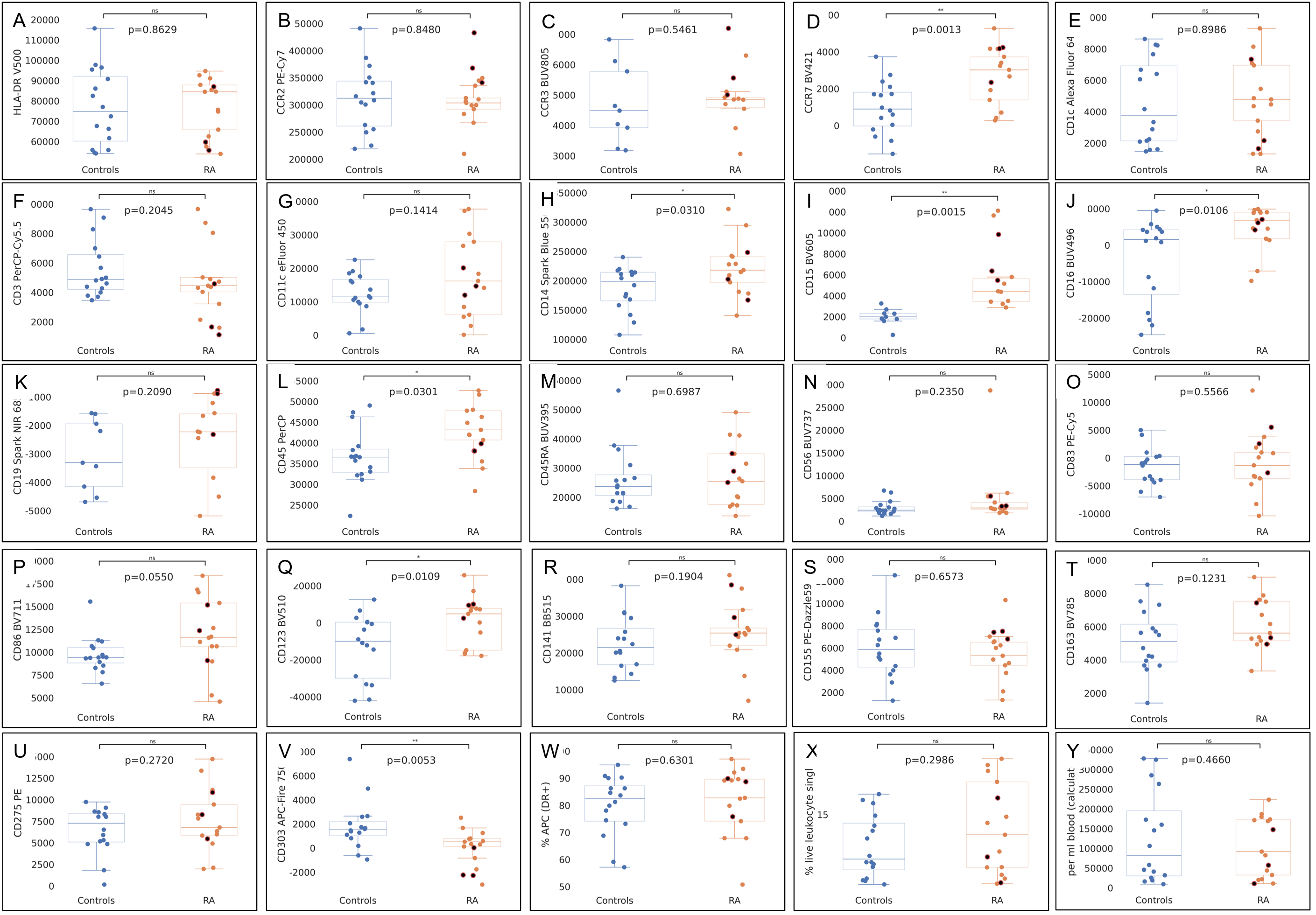


**Supplementary Figure 17**. Immunophenotyping results of CD14hiCD16lo monocytes (classical) of RA (orange) and healthy control donors (blue). (A) HLA-DR V500 (B) CCR2 PE-Cy7 (C) CCR3 BUV 805 (D) CCR7 BV421 (E) CD1c AF647 (F) CD3 PerCP-Cy5.5 (G) CD11c eFluor450 (H) CD14 SB550 (I) CD15 BV605 (J) CD16 BUV496 (K) CD19 Spark NIR 685 (L) CD45 PerCP (M) CD45RA BUV395 (N) CD56 BUV737 (O) CD83 PE-Cy5 (P) CD86 BV711 (Q) CD123 BV510 (R) CD141 BB515 (S) CD155 PE-Dazzle594 (T) CD163 BV785 (U) CD275 PE (V) CD303 APC-Fire 750. two-sided t-tests. * p<0.05 ** p<0.01 ***p<0.001


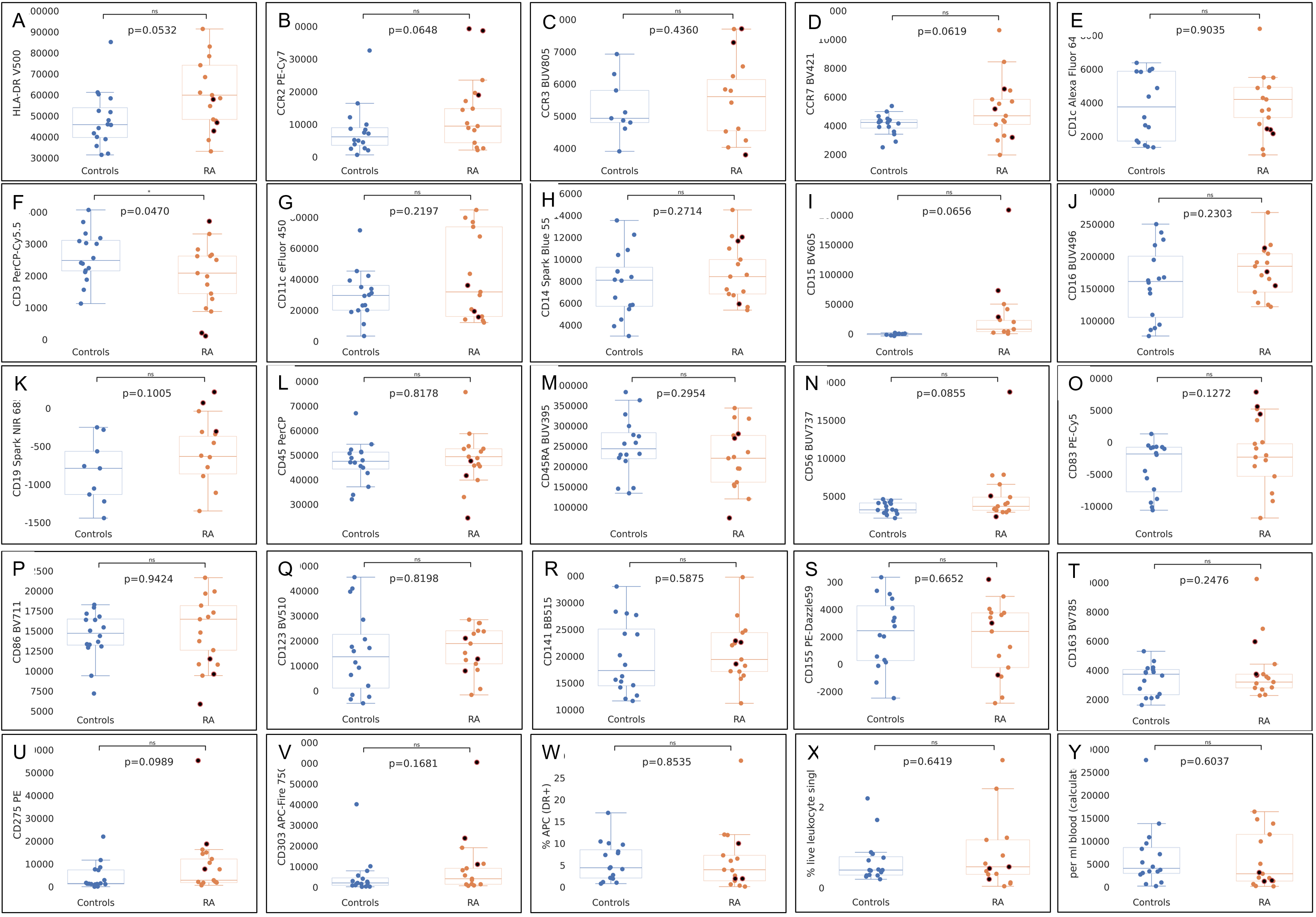


**Supplementary Figure 18**. Immunophenotyping results of CD14-CD16hi monocytes (non-classical) of RA (orange) and healthy control donors (blue). (A) HLA-DR V500 (B) CCR2 PE-Cy7 (C) CCR3 BUV 805 (D) CCR7 BV421 (E) CD1c AF647 (F) CD3 PerCP-Cy5.5 (G) CD11c eFluor450 (H) CD14 SB550 (I) CD15 BV605 (J) CD16 BUV496 (K) CD19 Spark NIR 685 (L) CD45 PerCP (M) CD45RA BUV395 (N) CD56 BUV737 (O) CD83 PE-Cy5 (P) CD86 BV711 (Q) CD123 BV510 (R) CD141 BB515 (S) CD155 PE-Dazzle594 (T) CD163 BV785 (U) CD275 PE (V) CD303 APC-Fire 750. two-sided t-tests. * p<0.05 ** p<0.01 ***p<0.001


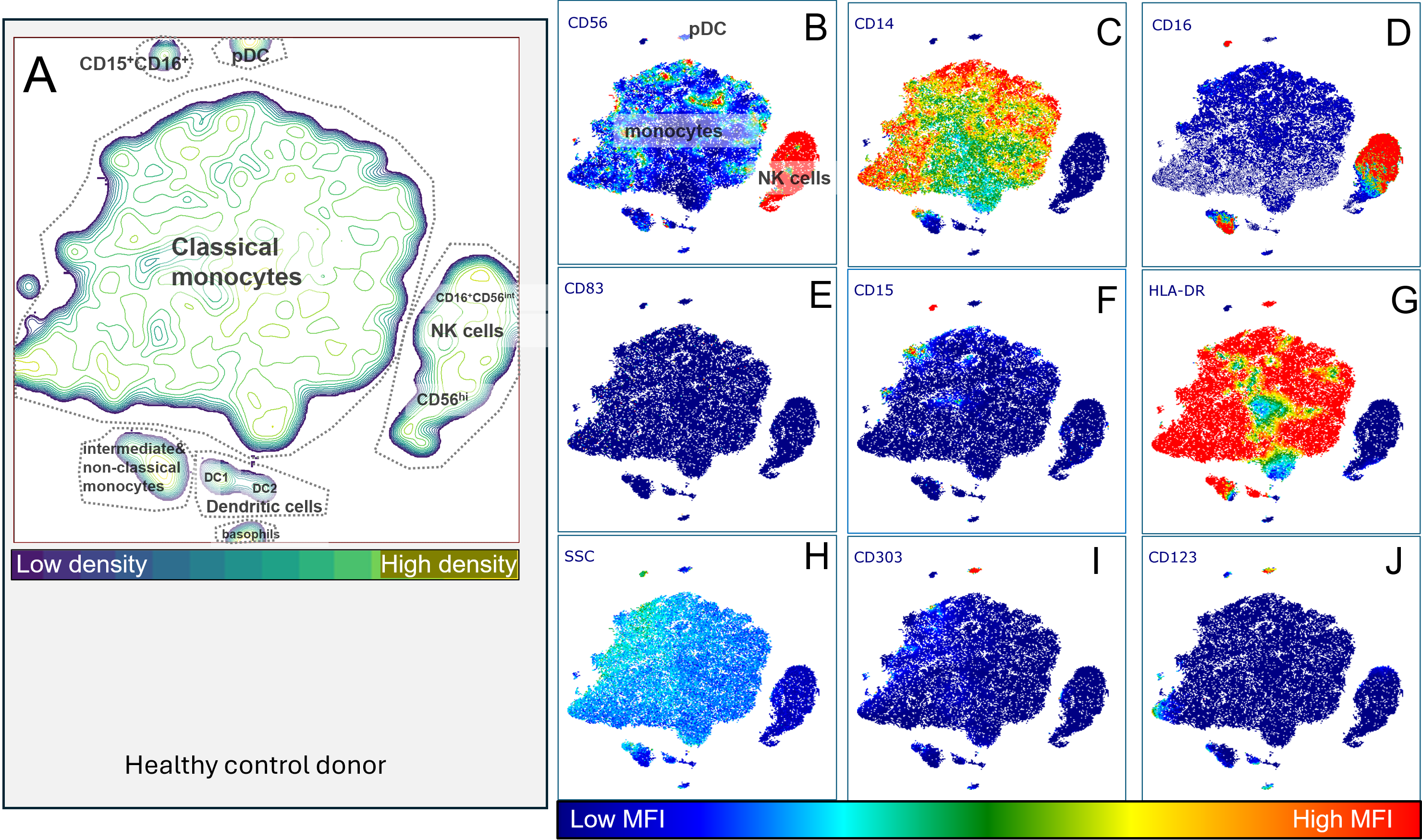


**Supplementary Figure 19.** t-SNE of APC (HLA-DR+) and CD56+ NK cells of a healthy control donor. Live singlet CD3-CD19- cells were gated and used for t-SNE. Annotated are the NK cell, dendritic cell and monocyte subsets.

*
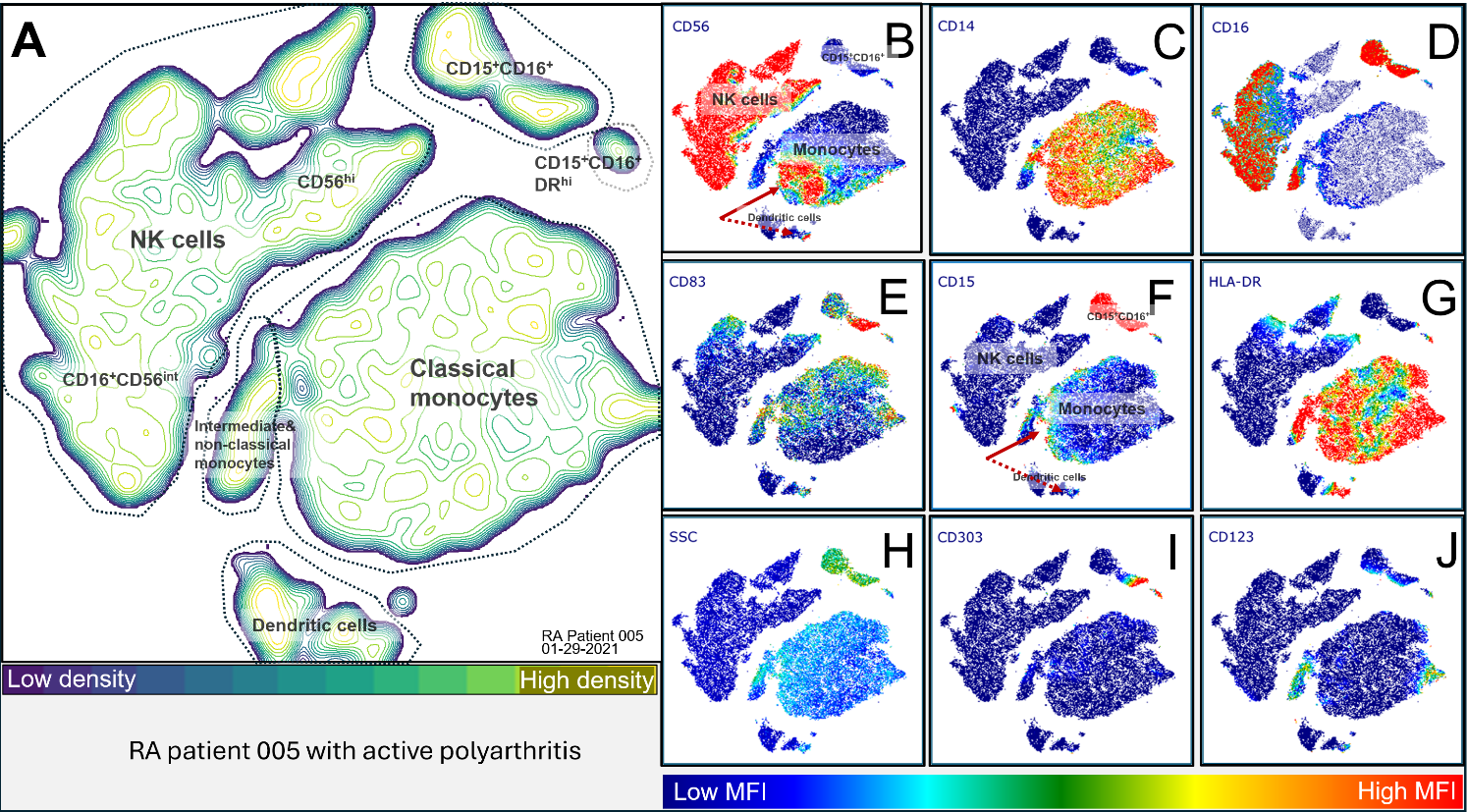
*

**Supplementary Figure 20.** t-SNE of APC (HLA-DR+) and CD56+ NK cells of an RA index patient. (A) Annotated cell populations (B) CD56 (C) CD14 (D) CD16 (E) CD83 (F) CD15 (G) HLA-DR (H) Side Scatter (SSC) (I) CD303 (J) CD123. Live singlet CD3-CD19- cells were gated and used for t-SNE. Annotated are the NK cell, dendritic cell and monocyte subsets.


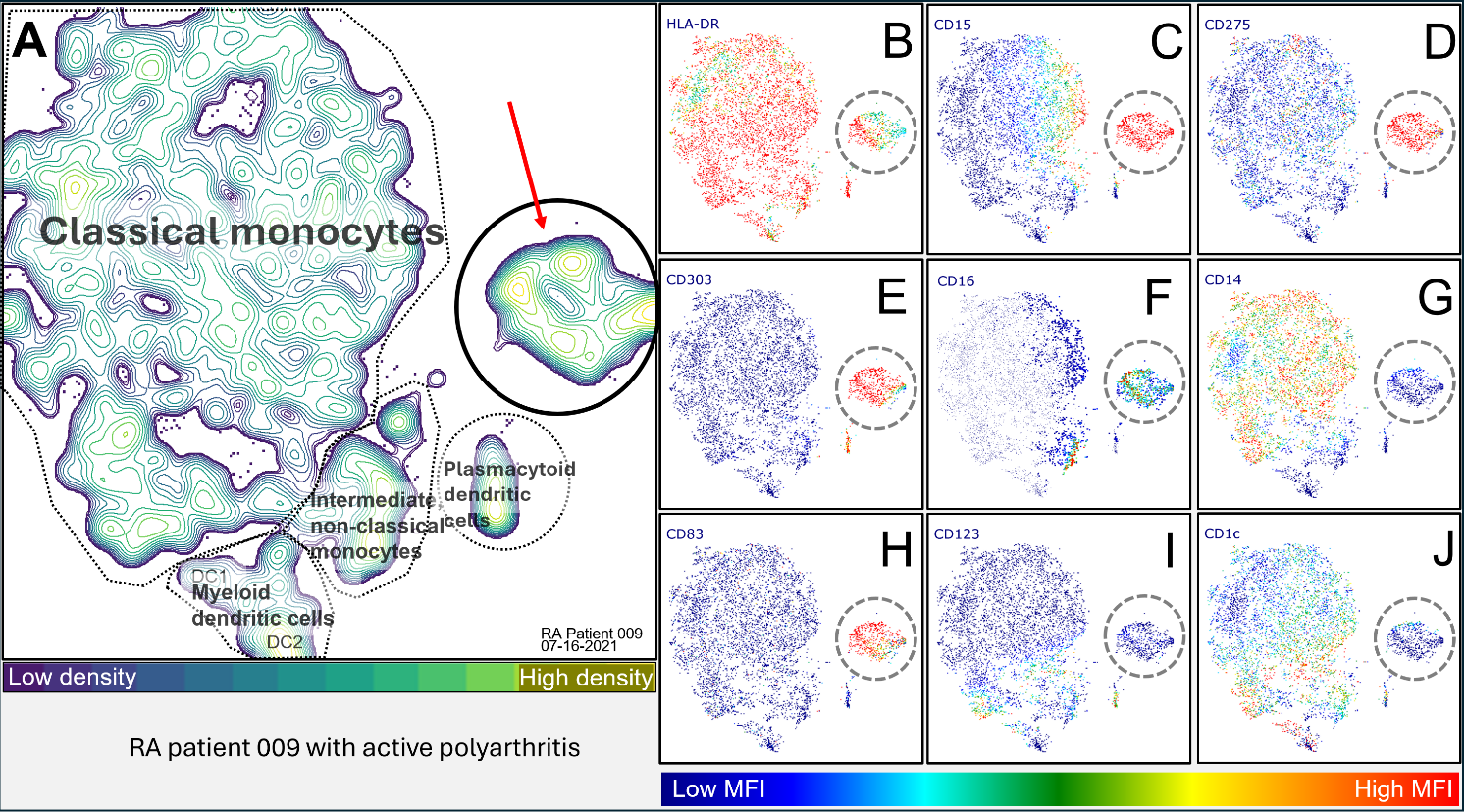


**Supplementary Figure 21.** t-SNE of antigen-presenting cells (HLA-DR+CD3-CD19-) of a RA index patient. (A) Annotated APC populations (B) HLA-DR (C) CD15 (D) CD275 (E) CD303 (F) CD16 (G) CD14 (H) CD83 (I) CD123 (J) CD1c. Annotated are the main dendritic cell and monocyte subsets. Live singlet HLA-DR+CD3-CD19- cells were gated and used for t-SNE. A distinct CD15+CD16+CD303+ population is marked separately (dashed ellipse, red arrow).


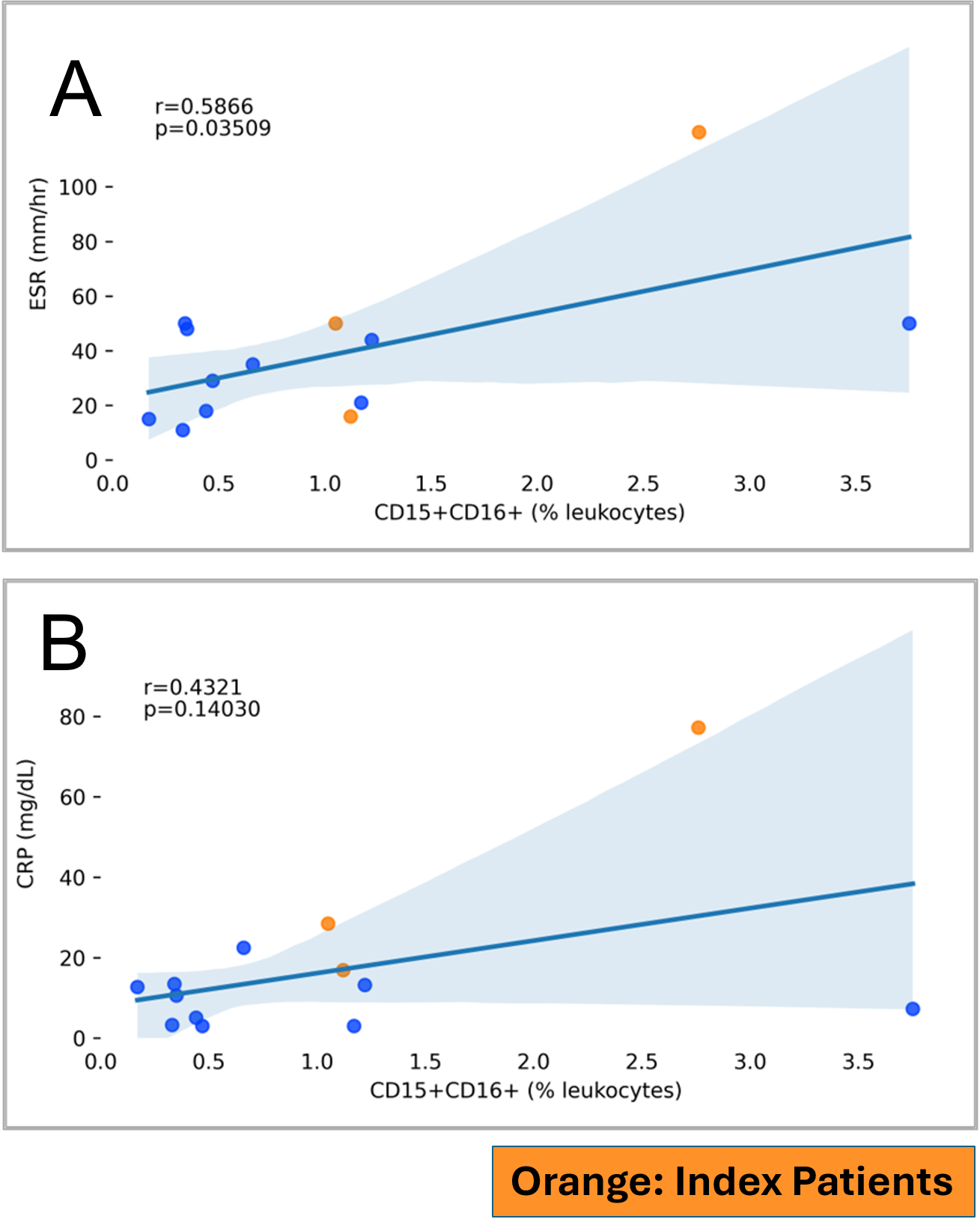


**Supplementary Figure 22**. Correlation of CD15+CD16+ (% leukocytes) and markers of inflammation in RA patients. RA index patients are highlighted separately. (A) ESR: Erythrocyte sedimentation rate (B) CRP: C-reactive protein.

## Supplementary Tables

|  | RA (n=16) | Healthy Controls (n=16) |
| --- | --- | --- |
| Age (mean, SD) | 54.6 ± 16.2 years | 54.0 ± 10.6 years |
| Gender (%, n) |  |  |
| Female | 75% (12) | 81.3% (13) |
| Male | 25% (4) | 18.8% (3) |
| Other | 0% (0) | 0% (0) |
| Ethnicity (%, n) |  |  |
| White | 75% (12) | 93.8% (15) |
| Black | 12.5% (2) | 0% (0) |
| Native American | 12.5% (2) | 0% (0) |
| South Indian | 0% (0) | 6.3% (1) |
| RA characteristics |  |  |
| RA duration since diagnosis (median [IQR]) | 4.4 [0.3 – 11.1] years | N/A |
| RA duration since initial symptoms (median [IQR]) | 5.4 [1.9 – 14.5] years | N/A |
| Rheumatoid Factor positive (%, n) | 100% (16) | N/A |
| Anti-CCP antibody positive (%, n) | 93.8% (15) | N/A |
| Treatment naïve (%, n) | 18.8% (3) | N/A |
| TNF inhibitor naïve (%, n) | 62.5% (10) | N/A |

**Supplementary Table 1**. Characteristics of RA patients and healthy control donors. Totals may not add up to 100% due to rounding.

| Patient ID | 005 | 009 | 013 |
| --- | --- | --- | --- |
| Age | 37 | 59 | 78 |
| Gender | Female | Male | Female |
| Ethnicity | White | White | White |
| Rheumatoid Factor | 74 IU/ml | 197 IU/ml | negative |
| CCP | greater than 250 U/ml | greater than 250 U/ml | 125 U/ml |
| Smoking status | never | smoker | never |
| Periodontal disease | No | No | No |
| Symptomatic Joints | Bilateral ankles and knees, bilateral wrists, MCP and PIP joints | Bilateral feet, bilateral ankles, bilateral knees, bilateral wrists, all MCP and PIP joints | Bilateral wrists, all MCP and PIP joints |
| RA treatment | Oral methotrexate, prednisone taper | Oral methotrexate, prednisone taper | Oral methotrexate, prednisone taper |
| Initial CRP | 28.5 mg/L | 77.2 mg/L | 52.9 mg/L |
| Initial ESR | 50 mm/hr | greater than 120 mm/hr | 40 mm/hr |
| RA symptom duration | 2 months | 3 years | 3 months |
| Time since RA diagnosis | 1 week | 1 week | 1 week |

**Supplementary Table 2**. Characteristics of RA ‘index’ patients with newly diagnosed debilitating disease. CCP: cyclic citrullinated polypeptide, CRP: C-reactive protein, ESR: Erythrocyte sedimentation rate

| Antibody (clone) | Fluorochrome | Vendor | Isotype | Catalog no. |
| --- | --- | --- | --- | --- |
| HLA-DR (G46-6) | V500 | BD Biosciences | Mouse IgG2a, κ | 561224 |
| CD16 (3G8) | BUV-496 | BD Biosciences | Mouse IgG1, κ | 612944 |
| CD14 (63D3) | SB550 | Biolegend | Mouse IgG1, κ | 367148 |
| CD56 (NCAM16.2) | BUV737 | BD Biosciences | Mouse IgG1, κ | 748609 |
| CD141 (1A4) | BB515 | BD Biosciences | Mouse IgG1, κ | 565084 |
| CD1c (L161) | AF647 | Biolegend | Mouse IgG1, κ | 331510 |
| CD123 (6H6) | BV510 | BD Biosciences | Mouse IgG1, κ | 751831 |
| CD303 (201A) | APCFire750 | Biolegend | Mouse IgG2a, κ | 354236 |
| CD45RA (HI100) | BUV395 | BD Biosciences | Mouse IgG2b, κ | 740298 |
| CCR2 (K036C2) | PE-Cy7 | Biolegend | Mouse IgG2a, κ | 357212 |
| CD11c (3.9) | eFluor450 | Invitrogen | Mouse IgG1, κ | 48-0116-42 |
| CD45 (2D1) | PerCP | Biolegend | Mouse IgG1, κ | 368506 |
| CD83 (HB15e) | PE-Cy5 | Biolegend | Mouse IgG1, κ | 305310 |
| CD86 (IT2.2) | BV711 | Biolegend | Mouse IgG2b, κ | 305440 |
| CCR7 (G043H7) | BV421 | Biolegend | Mouse IgG2a, κ | 353208 |
| CD155 (PVR) (SKII.4) | PE-Dazzle594 | Biolegend | Mouse IgG1, κ | 337616 |
| CD275 (ICOS-L) (2D3) | PE | Biolegend | Mouse IgG2b, κ | 309404 |
| CD163 (GHI/61) | BV785 | Biolegend | Mouse IgG1, κ | 333632 |
| CD15 (W6D3) | BV605 | BD Biosciences | Mouse IgG1, κ | 663987 |
| CCR3 (5E8) | BUV805 | BD Biosciences | Mouse IgG2b, κ | 749025 |
| CD3 (SK7) | PerCP5.5 | Biolegend | Mouse IgG1, κ | 344808 |
| CD19 (HIB19) | Spark NIR 685 | Biolegend | Mouse IgG1, κ | 302270 |
| Viability | Propidium iodide | Sigma-Aldrich | N/A | 537059 |
|  | Live/Dead Blue | ThermoFisher | N/A | L23105 |

**Supplementary Table 3**. Antibodies and Fluorochromes used for spectral flow cytometry.

| **Cell subset** | **Gating definition** | **Referred to as** |
| --- | --- | --- |
| CD141+ cDC1 | CD3-CD19- CD14lo/intCD16lo/int CD141+ live singlets | **DC1** |
| CD1c+ cDC2 | CD3-CD19- CD14lo/intCD16lo/int CD1c+ live singlets | **DC2** |
| CD163+ cDC3 | CD3-CD19- CD14lo/intCD16lo/int CD163+CD1c+ live singlets | **DC3** |
| CD123+CD303+ plasmacytoid DC | CD3-CD19- CD14lo/intCD16lo/int CD123+CD303+ live singlets | **pDC** |
| Non-lymphoid potential antigen-presenting cells | CD3-CD19- HLA-DR+ live singlets | **APC** |
| CD14hi CD16lo classical monocytes | CD3-CD19- CD14hi CD16lo live singlets | **Classical monocytes** |
| CD14hiCD16int intermediate monocytes | CD3-CD19- CD14hiCD16int live singlets | **Intermediate monocytes** |
| CD16hiCD14lo non-classical monocytes | CD3-CD19- CD16hiCD14lo live singlets | **Non-classical monocytes** |

**Supplementary Table 4** Summary of gating definitions and terminology for cell subsets.

## Supplementary datasets

Online repository with Python scripts and flow cytometry data in *.csv format: <https://github.com/christian-geier/apc-data>
